# Guadecitabine increases response to combined anti-CTLA-4 and anti-PD-1 treatment in mouse melanoma in vivo by controlling T-cells, myeloid derived suppressor and NK cells

**DOI:** 10.1186/s13046-023-02628-x

**Published:** 2023-03-18

**Authors:** Adriana Amaro, Francesco Reggiani, Daniela Fenoglio, Rosaria Gangemi, Anna Tosi, Alessia Parodi, Barbara Banelli, Valentina Rigo, Luca Mastracci, Federica Grillo, Alessandra Cereghetti, Aizhan Tastanova, Adhideb Ghosh, Fabio Sallustio, Laura Emionite, Antonio Daga, Tiziana Altosole, Gilberto Filaci, Antonio Rosato, Mitchell Levesque, Michele Maio, Ulrich Pfeffer, Michela Croce, EPigenetic Immune-oncology Consortium Airc (EPICA) consortium

**Affiliations:** 1 IRCCS Ospedale Policlinico San Martino, Largo Rosanna Benzi, 10, 16132 Genova, Italy; 2 Department of Internal Medicine, University of Genova, Genova, Italy; 3 Immunology and Molecular Oncology Diagnostics, Istituto Oncologico Veneto IRCCS, Padova, Italy; 4 Department of Dermatology, University of Zurich, University Hospital of Zurich, Zurich, Switzerland; 5 Functional Genomics Center Zurich, University of Zurich and ETH Zurich, Zurich, Switzerland; 6 Department of Interdisciplinary Medicine, University of Bari “Aldo Moro”, Bari, Italy; 7 Department of Surgery, Oncology and Gastroenterology, University of Padova, Padova, Italy; 8 University of Siena, Siena, Italy

**Keywords:** Melanoma, Guadecitabine, Anti-PD-1, Anti-CTLA-4, Tumor microenvironment, Treg, MDSC

## Abstract

**Background:**

The combination of Programmed Cell Death 1 (PD-1) and Cytotoxic T-Lymphocyte Antigen 4 (CTLA-4) blockade has dramatically improved the overall survival rate for malignant melanoma. Immune checkpoint blockers (ICBs) limit the tumor’s immune escape yet only for approximately a third of all tumors and, in most cases, for a limited amount of time. Several approaches to overcome resistance to ICBs are being investigated among which the addition of epigenetic drugs that are expected to act on both immune and tumor cells. Guadecitabine, a dinucleotide prodrug of a decitabine linked via phosphodiester bond to a guanosine, showed promising results in the phase-1 clinical trial, NIBIT-M4 (NCT02608437).

**Methods:**

We used the syngeneic B16F10 murine melanoma model to study the effects of immune checkpoint blocking antibodies against CTLA-4 and PD-1 in combination, with and without the addition of Guadecitabine. We comprehensively characterized the tumor’s and the host’s responses under different treatments by flow cytometry, multiplex immunofluorescence and methylation analysis.

**Results:**

In combination with ICBs, Guadecitabine significantly reduced subcutaneous tumor growth as well as metastases formation compared to ICBs and Guadecitabine treatment. In particular, Guadecitabine greatly enhanced the efficacy of combined ICBs by increasing effector memory CD8+ T cells, inducing effector NK cells in the spleen and reducing tumor infiltrating regulatory T cells and myeloid derived suppressor cells (MDSC), in the tumor microenvironment (TME). Guadecitabine in association with ICBs increased serum levels of IFN-γ and IFN-γ-induced chemokines with anti-angiogenic activity. Guadecitabine led to a general DNA-demethylation, in particular of sites of intermediate methylation levels.

**Conclusions:**

These results indicate Guadecitabine as a promising epigenetic drug to be added to ICBs therapy.

**Supplementary Information:**

The online version contains supplementary material available at 10.1186/s13046-023-02628-x.

## Background

The combination of Programmed Cell Death 1 (PD-1) and Cytotoxic T-Lymphocyte Antigen 4 (CTLA-4) blockade determined an improved overall survival rate at 3 years of 58% in clinical trials as compared to ipilimumab alone [1, 2] and this corresponds to the real world experience [3, 4]. Indeed, this treatment has been approved for unresectable or metastatic melanoma. Immune checkpoint blockers (ICBs) limit the tumor’s immune escape yet only for approximately a third of all tumors and, in most cases, for a limited amount of time. Resistance to ICBs can be primary for tumors that are intrinsically “invisible” by the immune system, adaptive for tumors that are recognized by the immune system but adapt to it, and truly acquired for tumors that initially respond to the treatment but then progress [5] reminiscent of cancer immune-editing theory [6]. Pre-requisite of tumor response to ICBs is the co-expression by cancer cells of immunogenic tumor antigens and targetable immune checkpoint molecules. Several approaches to overcome all types of resistance to ICBs are being investigated among which the addition of epigenetic drugs that are expected to act on both, immune and tumor cells [7–9]. In order to be effective, epigenetic therapy is expected to exert several of the following functions: 1) to recover/induce tumor neo-antigens presentation [10]; 2) to favor recruitment of antigen–specific cytotoxic CD8+ T cells into the tumor microenvironment (TME) [11, 12]; 3) to restore activating co-stimulatory molecular pathways [13] counteracting T-cell exhaustion [14, 15]; 4) to reduce tumor infiltration by immune regulatory T-cells [16, 17] and myeloid derived suppressor cells (MDSC) [18, 19]. Early clinical trials sustain the concept of epigenetic enhancement of immunotherapy [20, 21] yet the precise mechanism of action and the crucial cellular targets of epigenetic drugs remain unknown.

Guadecitabine is a dinucleotide prodrug of a decitabine linked via phosphodiester bond to a guanosine. Upon metabolic activation by phosphorylation and incorporation into DNA, guadecitabine inhibits DNA Methyltransferase 1 (DNMT1), thereby causing non-specific hypomethylation. Guadecitabine is resistant to cytidine deaminase and gradually releases decitabine leading to a more prolonged exposure to the active drug [22]. Guadecitabine is among epigenetic drugs provided with remarkable immune modulating activities [23–26]: for this reason it may be a valid candidate to be co-administered with ICBs. Anichini et al. compared the effects of several epigenetic drugs targeting histone deacetylase (HDAC), polycomb repressive complex (PRC) and bromodomain and extraterminal protein (BRD) to those elicited by guadecitabine in primary melanoma cells. Considering immune-activating and -repressive functions, guadecitabine appeared the most potent immunomodulatory epigenetic drug [27].

Based on this, we decided to explore its effects on TME and the host’s immune responses when associated with CTLA-4 and PD-1 ICBs (administered alone or in combination). The study was performed in the experimental syngeneic B16F10 murine melanoma model, which is widely used since it recapitulates salient features of human melanoma [28]: notably, the first indication of efficacy by PD-1 blockade in controlling malignant melanoma was obtained in this model [29].

The analysis of the effect of combining anti-CTLA-4 and anti-PD-1 antibodies with guadecitabine presented here anticipates the expected results of an ongoing clinical phase-II trial using this combination in melanoma and lung cancer patients who are resistant to anti-PD-1/-PD-L1 therapy (NCT04250246) [30]. We show here, that guadecitabine leads to a general DNA-demethylation and greatly enhances the efficacy of combined ICBs by affecting tumor infiltrating regulatory T cells, myeloid derived suppressor cells (MDSC) and macrophages and by inducing T and NK cytotoxic responses.

## Methods

### Cell lines and reagents

B16F10 (ATL99010) mouse melanoma cell line was purchased from ICLC (Genoa, Italy; authentication by institutional biological banking facility using STR according to International Cell Line Authentication Committee (ICLAC) guidelines). Cells are grown in DMEM supplemented with 10% FBS, Glutamine and antibiotics (Life Technologies Corporation, San Francisco, CA, USA).

Guadecitabine (HY-15229, MedChem Express, Sollentuna, Sweden) powder was resuspended in water at the concentration of 10mM. For *in vivo* treatment 1mg/kg guadecitabine (modified from [31]) was diluted in PBS and given to mice by Intraperitoneal (IP) injections to a final volume of 0.1 ml/mouse from day 3 to 16, daily, post sub cutaneous (SC) injection of cancer cells, or from day +1 to +15 days, daily, post intravenous (IV) challenge. Vehicle (PBS) was used for control mice given at the same time points as guadecitabine.

InVivoPlus anti-mouse CTLA-4 (CD152), [9H10], and InVivoPlus anti-mouse PD-1 (CD279), [RMP1-14], and isotype controls (InVivoPlus rat IgG2a isotype control, [2A3] and/or InVivoPlus polyclonal Syrian hamster IgG, [Polyclonal Syrian]), (BioXcell,West Lebanon, NH, USA), each at 200 μg/mouse/dose, were given IP at days: +4, +7, +10, +13, +16 (SC model) or +2, +5, +8, +11, +14 (pseudo-metastatic model) diluted in InVivoPure pH 7.0 Dilution Buffer (BioXcell). Anti-CTLA4 and anti-PD1 were administered with a similar schedule of Wang et al. [32].

### Animal model

Eight-week-old C57black/6J mice were purchased from Charles River (Charles River Laboratories, Milan, Italy). The animals were housed in pathogen-free colony, and experiments were performed in under the National Regulation on Animal Research Resources and approved by the Review Board of the IRCCS Ospedale Policlinico San Martino, Genoa, and Italian Ministry of Health (n°74/2020-PR released on 05/02/2020, according to art.31 legislative decree 26/2014). Mice were shaved, and injected SC in the right flank with 10^5^ B16F10-luc (>90% viable) in a volume of 0.1 ml serum-free medium. Tumor volume was calculated as follows: V= ½ x L x W x H. For the pseudo-metastatic model 4x10^5^ B16F10 cells were injected through the tail vein in a volume of 0.1 ml serum-free medium. After the injection of tumor cells, mice were randomly separated in groups of 5-10 animals/group. To evaluate distress in response to treatment we monitored mice twice a week from the beginning of the treatments and focused on: altered or impaired gait, reduced coordination of movements, reduced reactivity, dehydration, emaciation, neurological signs or a significant reduction (>15%) of body weight. Mice were sacrificed by CO2 asphyxiation when their tumor masses reached 1 cm^3^ or any other sign of disease. In the pseudo-metastatic model mice were sacrificed the day after the end of treatments. At the time of sacrifice, blood, spleen and lymph nodes were taken from treated and Ctrl mice. Tumors were divided in two: one part for immunofluorescence analysis and one part was formalin-fixed paraffin-embedded (FFPE) for further analyses. Blood, spleen, tumor and lymph node were used freshly or stored at -80°C or nitrogen.

### Immunofluorescence analysis

Immunofluorescence analyses were performed on fresh and frozen samples (tumors, spleen and lymph nodes) from both treated and control mice. Tumors are smashed using a 70micron FALCON-cell strainer (Corning, Merck Life Science srl, Milan, Italy), cell suspension is counted and incubated with specific fluorochrome-conjugated monoclonal antibodies (mAbs) at 4 °C for 30 min in the dark. IFN-γ was detected previous standard incubation with PMA, ionomycin and monensin for 4hrs at 37°c. The antibodies used, catalogue number and the supplier company are listed in Table 1. To perform intranuclear and intracellular staining, surface stained cells were fixed and permeabilized with Fix/Perm Buffer Set (Biolegend, Amsterdam, The Netherlands) according to manufacturer’s instructions in the dark with the fluorochrome‐conjugated anti‐FoxP3 (BD Biosciences, Milan, Italy) or anti-GranzymeB (Biolegend). The cells were washed with 1 ml of phosphate‐buffered saline–bovine serum albumin (PBS‐BSA) 0.01% and resuspended in 300 μl of PBS. The samples were analyzed by a BD Fortessa X20 flow cytometer (BD Biosciences) using the BD FACS Diva™ software version 8.0 (BD Biosciences) or FlowJo (Ashland, USA).

**Table 1:** List of antibodies

**Supplier**	**Cat. No.**	**Host**	**Antigen**	**Fluorochrome**
BD Biosciences	561827	hamster	**CD3**	FITC
BD Biosciences	562012	mouse	**I-Ab**	PE
BD Biosciences	553720	hamster	**CD152**	PE
BD Biosciences	561096	mouse	**CD45.2**	PerCPCy5.5
life technology	35-0114-82	hamster	**CD11c**	PECy5,5
BD Biosciences	552775	rat	**CD4**	PECy7
BD Biosciences	560593	rat	**Ly-6C**	PECy7
Biolegend	143810	rat	**CD39**	APC
life technology	17-6691-82	rat	**EGR2**	APC
BD Biosciences	564715	rat	**CD274 (PDL-1)**	APC
BD Biosciences	565135	rat	**CD25**	APC-R700
BD Biosciences	565815	hamster	**CD279 (PD-1)**	APC-R700
BD Biosciences	564985	rat	**CD11b**	APC-R700
Biolegend	372216	mouse	**Granzyme B**	PECF594
BD Biosciences	562700	rat	**Ly-6G**	PECF594
BD Biosciences	747626	mouse	**TIM-3**	BV421
BD Biosciences	566297	rat	**CD103**	BV421
BD Biosciences	562966	rat	**FoxP3**	BV421
BD Biosciences	746476	rat	**CD38**	BV480
BD Biosciences	563117	rat	**CD62L**	BV510
BD Biosciences	563058	rat	**CD44**	BV605
BD Biosciences	564069	rat	**NKp46**	BV605
BD Biosciences	747623	mouse	**TIM-3**	BV650
BD Biosciences	740466	hamster	**CD28**	BV650
BD Biosciences	745287	mouse	**H-2Kb**	BV650
BD Biosciences	563755	mouse	**Ki-67**	BV711
BD Biosciences	563157	rat	**CD19**	BV711
BD Biosciences	563332	rat	**CD8a**	BV786
BD Biosciences	564336	rat	**IFN-γ**	BV711
BD Biosciences	558661	rat	**CD107a**	PE
BD Biosciences	565388		**FVD**	APCH7

### Milliplex ELISA

Blood samples were collected from treated and control mice from both SC and IV model of melanoma at mice sacrifice. Serum samples were analyzed for cytokine and chemokine levels by Mouse cytokine/chemokine magnetic bead panel, Milliplex Map kit (MCYTOMAG-70K-PX32, Millipore, Billerica, MA, USA) accordingly to manufacturer procedures, using a Luminex MagPix reader with xPONENT software (Millipore). Quality controls were included to qualify assay performance and the concentration values respected their ranges.

### Multiplex Immunofluorescence (mIF)

The Tyramide Signal Amplification (TSA)-based Opal method (Akoya Biosciences, Marlborough, MA, USA) was used for mIF staining on the Leica BOND RX automated immunostainer (Leica Biosystems, Wetzlar, Germania). Prior to staining, all 4 µm-thick FFPE tissue sections were deparaffinised by baking over night at 56 °C, soaking in BOND Dewax Solution at 72 °C, and then rehydrating in ethanol. Heat-induced epitope retrieval (HIER) pretreatments were applied at 97 °C using BOND Epitope Retrieval (ER) Solutions: citrate-based pH 6.0 ER1 or EDTA-based pH 9.0 ER2 (both Leica Biosystems, Wetzlar, Germania). Tissue sections were blocked with Normal Goat Serum (Vector Laboratories, Newark, CA, USA) for 10 minutes before applying each primary antibody. Before proceeding with multiplex staining, a fluorescent singleplex was carried out for each biomarker to determine the optimal staining conditions and the order in which the primary antibodies would be applied in the multiplex protocol. The following primary antibodies were added sequentially on the slides: rat anti-mouse F4/80 (clone CI:A3-1, Bio-Rad, Hercules, CA, USA), rat anti-mouse Ly6C (clone ER-MP20, Abcam, Cambridge, UK), rabbit anti-mouse Ly6G (clone EPR22909-135, Abcam), rat anti-mouse CD8a (4SM15, Thermo Fisher, Waltham, MA, USA), rat anti-mouse FoxP3 (clone FJK-16S, Thermo Fisher), rat anti-mouse CD4 (clone 4SM95, Thermo Fisher,), rabbit anti-mouse CD206 (clone E6T5J, Cell Signaling Technologies, Danvers, MA, USA), rabbit anti-mouse Melan-a (clone EPR20380, Abcam). The HRP-conjugated secondary antibodies goat anti-rabbit and goat anti-rat (both Vector Laboratories) were incubated as appropriated for 10 minutes. The TSA-conjugated Opal fluorophores (Akoya Biosciences) were then added for 10 minutes. Slides were rinsed with washing buffer after each step. Finally, the spectral DAPI (Akoya Biosciences) was used as nuclear counterstain, and slides were mounted in ProLong Diamond Anti-fade Mountant (Life Technologies).

Multiplex stained slides were imaged using the Mantra Quantitative Pathology Workstation (Akoya Biosciences) at 20X magnification. For each sample, only areas comprising tumor cells were considered. The inForm Image Analysis software (version 2.4.9, Akoya Biosciences) was used to unmix multispectral images. A selection of representative multispectral images was used to train the inForm software to create algorithms to apply in the batch analysis of all acquired multispectral images. phenoptrReports (add-ins for R Studio from Akoya Biosciences) was used to calculate cell density and spatial metrics. Cell density data were calculated as the sum of the cells positive for a specific marker, divided by the area analyzed from the same tissue slide. Cell density and cell percentage results refer to the total area analyzed (tumor plus stroma), the intra-tumoral area only or the peri-tumoral stroma only, as indicated. For mean distance between different cell subtypes, the nearest neighbour analysis was used, while the count within analysis was employed to calculate the number of reference cells that are present within a 30 µm radius from a cell with a different phenotype, and normalized for the total number of reference cells.

### Methylome array analysis

Bisulfite conversion of DNA extracted from mice tissues was performed with the EZ-96 DNA Methylation Kit (Zymo Research, Irvine, CA, USA), and subsequent hybridization of this DNA was carried out on the Illumina Infinium Mouse Methylation BeadChip (Illumina, San Diego, CA, USA).

The chip interrogates DNA methylation at more than 285000 CpGs, provides balanced coverage of CpG islands, transcription start sites (TSS), enhancers, imprinted loci, gene body regions, repetitive element regions, lamin attachment domains, CTCF binding sites, and hypermethylated regions in cancer. The DNA methylation score at each CpG, described as the DNA methylation β value, ranges between 0 and 1 and is derived from the fluorescent intensity ratio (β = intensity of the methylated allele ÷ (intensity of the unmethylated allele + intensity of the methylated allele + 100)). DNA-methylation microarray data are available under accession number GSE220698 at the GEO (http://www.ncbi.nlm.nih.gov/geo/). All statistical analysis was performed using R and the RnBeads R package [33]. First, a prefiltering step was performed to remove SNP-enriched probes that overlap with more than 2 SNPs. Then, the Greedycut algorithm was applied. It iteratively removes probes and samples of highest impurity from the dataset. We analyzed more than 285000 CpGs targeted across genes, promoters, 5’-untranslated regions (UTRs), first exons, gene bodies and 3’UTRs.

One thousand six hundred seventy-one CpG sites were removed from the analysis because they overlapped with SNP-enriched regions. Moreover, we filtered out 2374 probes that contained the largest fraction of unreliable measurements when the Greedycut algorithm was applied. We considered every β value to be unreliable when its corresponding detection *p*-value was not below the threshold T: p ≥ T = 0.05. Normalization was then applied to the data. The background was subtracted using the methylumi package (method “noob”) [34]. The signal intensity values were normalized using the SWAN normalization method, as implemented in the minfi package [35].

Differential methylation analysis on site and region level was computed considering the difference in mean methylation levels of mice treated with ICBs alone or triple therapy, the quotient in mean methylation and the t-test assessing whether the methylation values in the two groups originate from distinct distributions. Additionally, each site was assigned a rank based on each of these three criteria. A combined rank was computed as the maximum (i.e. worst) rank among the three ranks. The 1% most variable probes according to the MAD index (Median Absolute Deviation) were selected for unsupervised analysis with R, as previously applied to Skin Cutaneous Melanoma methylation array data [36]. All samples and selected features were clustered as reported in the heatmap of Fig. 13, produced by the Pheatmap package [37] and for the pathway analysis. To assess biological relationships among differently methylated DNA regions/genes, the Ingenuity Pathway Analysis software was used (IPA, Ingenuity System, Qiagen, Germantown, MD, USA). IPA generates networks based on the connectivity of the genes and computes a score for each network according to the fit of the set of supplied focus regions. These scores indicate the likelihood of focus genes belonging to a network versus those obtained by chance. The canonical pathways generated by IPA are the most significant for the uploaded data set. Fischer’s exact test with the FDR option was used to calculate the significance of the canonical pathway.

### Statistical analyses

All the comparisons between control and treated mice were evaluated using two-sided T-test for independent samples or two-way ANOVA. *P* values lower than 0.05 were considered as significant. *P* values are shown as follows: (*p<0.05, **p<0.02, ***p<0.01, ****p<0.001). Statistical analyses were performed using PRISM 9.4 (Graph-Pad Software, San Diego, CA, USA).

## Results

### Guadecitabine reduces B16F10 tumor growth in vivo

**Fig. 1:**
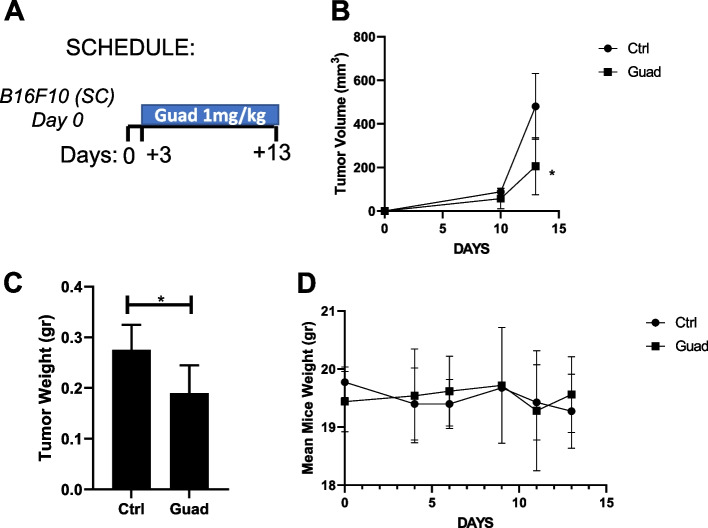
Guadecitabine continuous treatment significantly reduces tumor growth. **A** schedule of treatments. **B** tumor volume reduction in mice treated with 1mg/kg guadecitabine (Guad) for 13 consecutive days (Day +13 *p*<0.01). *n*=5 mice/group. **C** significative reduction of mean tumor weight in mice receiving guadecitabine (Guad). *p<0.05. **D** no difference in mean mouse weight between control and treated mice

C57Black/6J mice, challenged SC with B16F10 cells, received daily treatment with guadecitabine (1mg/kg) or vehicle (PBS) starting 3 days post melanoma cell injection until day +13 (Fig. 1A), when mice were sacrificed, tumors excised and weighted. Continuous treatment with the drug significantly decreased mean tumor volume at day 13 (Fig. 1B). Accordingly, tumor weight was significantly lower in guadecitabine treated mice than in controls, supporting the evidence that continuous administration of low dose guadecitabine reduces tumor growth *in vivo* (Fig. 1C). No sign of evident toxicity was observed since no difference in the mean weight of the mice was detected between guadecitabine and the control group during treatment (Fig. 1D). In addition, no altered or impaired gait, reduced coordination of movements, reduced reactivity, dehydration, emaciation, neurological signs were detected in mice receiving guadecitabine.

### Guadecitabine in combination with anti-PD-1 and anti-CTLA-4 mAbs greatly reduces tumor growth in vivo

**Fig. 2:**
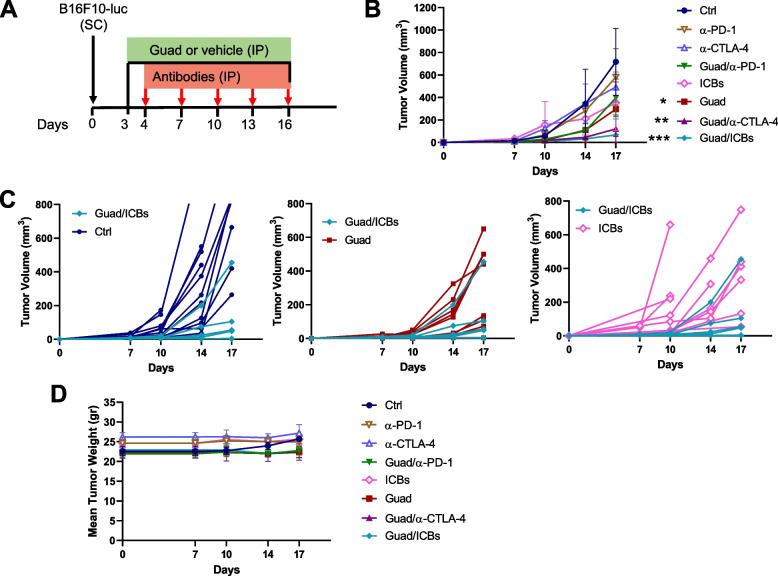
Triple therapy significantly reduces *in vivo* tumor growth. **A** schedule of treatments: B16F10 cells were injected SC on day 0 (black arrow), guadecitabine or vehicle were given IP daily from day +3 to day +16 (black line), antibodies (anti-CTLA-4 and/or anti-PD-1 or isotype controls) were given IP on days +4, +7, +10, +13, +16 (red arrow). **B** Guadecitabine significantly increased the anti-tumoral effects of anti-CTLA-4 and anti-PD-1 mAbs (ICBs). (*p<0.05; ***p<0.01, respect to control). **C** Spaghetti plots of the volume of the single tumors in mice treated with guad alone versus triple therapy (top panel), ICBs vs. triple therapy (middle panel) and control vs. triple therapy (bottom panel). Some curves overlap. **D** Mice weight in grams. No significative differences in weight were detected among different group of treatments. *n*=9 mice /group of treatment

We then assessed the effects of guadecitabine in association to anti-PD-1 and -CTLA-4 mAbs (ICBs). Mice were SC challenged with B16F10 cells. After three days, mice were randomized in eight groups that received daily treatment with guadecitabine (or vehicle) either alone or in association with anti-CTLA-4 and/or anti-PD-1 mAbs and with anti-CTLA-4 and/or anti-PD-1 mAbs or isotype controls (Fig. 2A, B). Tumor growth of mice who received guadecitabine/anti-CTLA-4/anti-PD-1 (guadecitabine/ICBs; triple therapy) was compared to control or ICBs treated mice.

Mice treated with guadecitabine/ICBs showed the most significant growth reduction at any day of measurement in comparison to the control group. Guadecitabine showed a greater effect when associated with anti-CTLA-4 than to anti-PD-1 mAbs suggesting that epigenetic modulation may preferentially synergize with blockade of a specific immune checkpoint (Fig. 2B). Yet triple therapy showed a stronger antitumor effect than either guadecitabine/anti-CTLA-4 (guad/α-CTLA-4), guadecitabine/anti-PD-1 (guad/α-PD-1) or guadecitabine alone (Fig. 2B). The analysis of the growth of single tumors (Fig. 2C) showed considerable variability without hiding the significant effects that the addition of guadecitabine had on the efficacy of ICBs. No macroscopic sign of toxicity was observed, since mean mice weights were similar between the different groups of treatment (Fig. 2D).

### Guadecitabine in combination with anti-PD-1 and anti-CTLA-4 mAbs reshapes TME to anti-tumor responsiveness

**Fig. 3:**
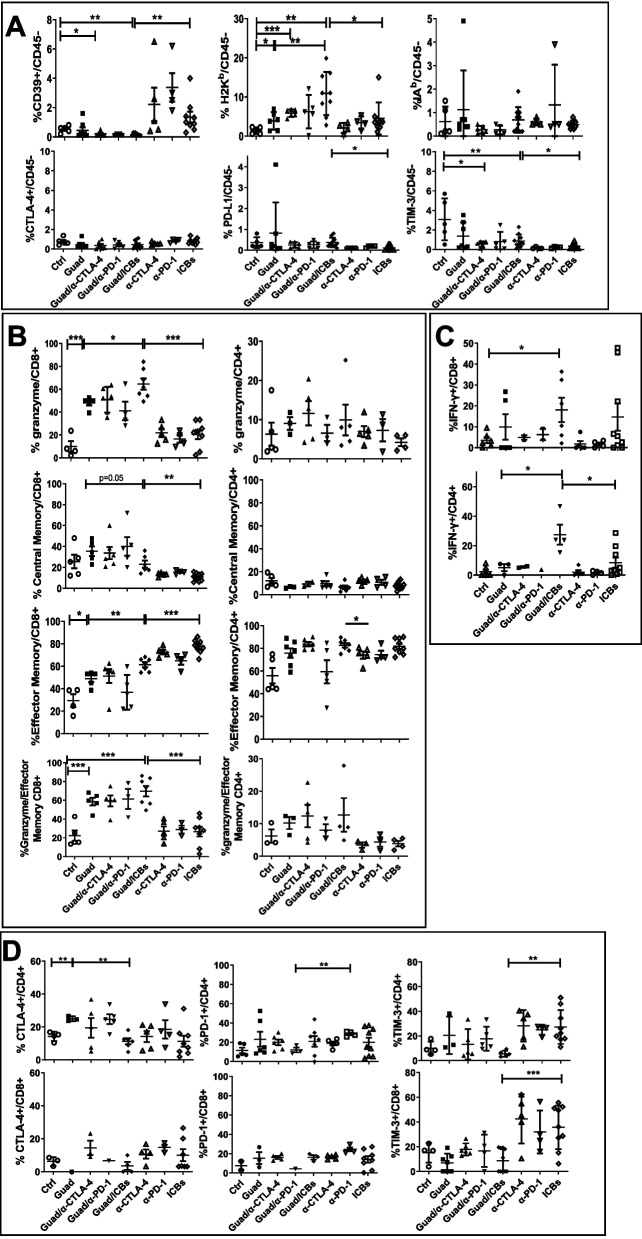
TME modifications induced by different treatments. Cell suspensions from tumors were analyzed by flow cytometry. **A** Analysis of CD39, MHC-class I (H2Kb), MHC-class II (IAb), CTLA-4, PD-L1 and TIM-3 expression on B16F10 cells (CD45neg). **B** expression of granzyme on CD8+ and CD4+ T cells, maturation of CD8+ and CD4+ T cells in central (CD44+CD62L+) and effector (CD44+CD62L-) memory cells and granzyme expression on effector memory CD8+ and CD4+ T cells. **C** IFN-γ expression on CD8+ and CD4+ T cells. **D** expression of CTLA-4, PD-1 and TIM-3 immune checkpoints on CD4+ and CD8+ T cells referred to CD3+. *p<0.05, **p<0.02, ***p<0.01

TME modifications induced by different treatments were analysed by flow cytometry on cell suspensions recovered from tumors. Guadecitabine deeply modified tumor cells within TME inducing the expression of MHC-class I, but not -class II molecules, compared to control, potentially enabling B16F10 cells to present antigens to CD8+ T cells (Fig. 3A). We found that ICBs increased CD39, an ectonucleotidase involved in the conversion of ATP to immunosuppressive adenosine [38, 39], on B16F10 melanoma cells but this effect was efficiently counteracted by guadecitabine. Notably, CD39 was found highly expressed by different tumors including melanoma [40, 41]. CD39 expression by tumor cells favors resistance to chemotherapy and immunotherapy so that trials testing the therapeutic efficacy of CD39 blockade, alone or in combination with ICBs, are under way [42].

In addition, compared to control, ICBs, with or without guadecitabine, downmodulated the expression of TIM-3, an alternative immune checkpoint, particularly expressed on B16F10 cells (Fig. 3A), similarly to what found on human melanoma cells [43]. CTLA-4 and PD-L1 expression on B16F10 cells was negligible in all tested conditions (Fig. 3A).

**Fig. 4:**
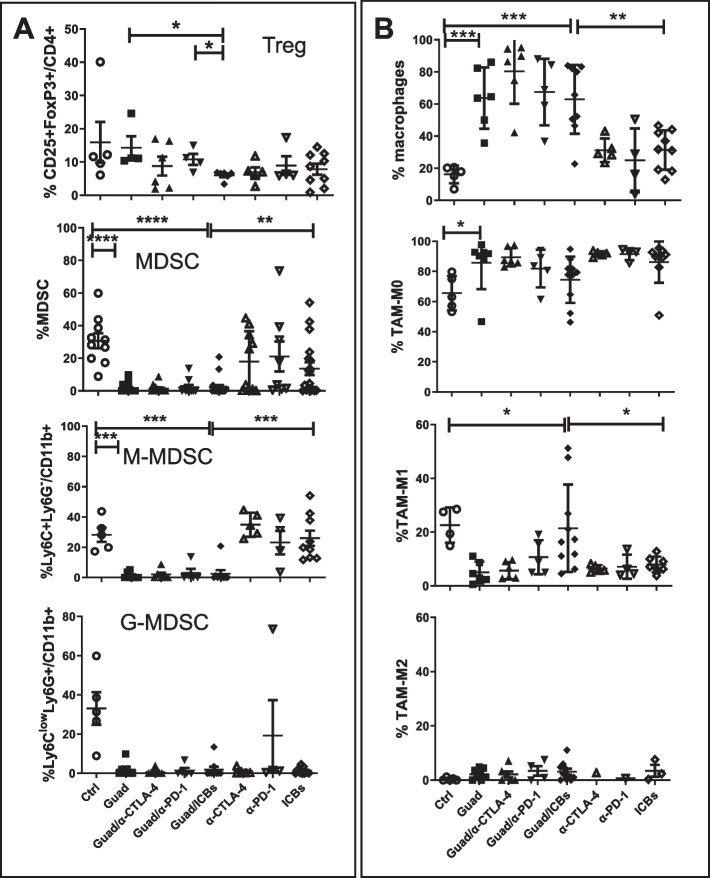
Guadecitabine modifies TME by increasing macrophages, while Guadecitabine/ICBs skews TAM to M1 subset, without upregulating alternative immune checkpoint. **A** identification of Treg (CD45+CD3+ CD4+CD25+Foxp3+), and MDSC (CD45+CD11b+Ly6C+/-Ly6G+/-) and subset M (Ly6C+Ly6G-) G (Ly6ClowLy6G+) on CD45+ cells infiltrating tumors. **B** frequencies of macrophages (Ly6C-Ly6G- on CD45+CD3-CD19-NKp46-I-A^b^-CD11c-/+) and identification of TAM subsets: M0 CD38-Egr2-, M1 CD38+Egr2-, M2 CD38-Egr2+. *p<0.05, **p<0.02, ***p<0.01

To study TME reshaping we also analyzed the inflammatory tumor infiltrate. Total T cells, as well as CD4+ and CD8+ T cell subset, frequencies were comparable among all the different groups of treatment (Supplementary Figs. 1A and 2A), although treatments containing guadecitabine showed a non-significant decrease in CD45+ cells, compared to ICBs. However, remarkable differences among groups were detected focusing on maturation stages and functions of CD8+ T cells. As compared to control or ICBs, guadecitabine alone and in combination with ICBs upregulated T cell responses by increasing granzyme production on tumor infiltrating CD8+ T cells, favoring the maturation of CD8+T cells in effector memory (CD44+CD62L-) cells secreting granzyme (Fig. 3B). These data suggest that guadecitabine/ICBs are able to unleash maturation pathways within CD8+ T cells committing these cells to effector populations. In this process guadecitabine seems to have a leading role since mice treated with this drug, but not those treated with ICBs alone, showed expansion of granzyme^+^ CD8+ T cells (i.e., effector T cells already prompted to cytotoxicity), whose frequency strictly paralleled that of effector memory CD8+ T cells (Fig. 3B). Although neither guadecitabine nor ICBs showed effects on maturation stage and acquisition of granzyme-related cytotoxic function by CD4+ T cells (Fig. 3B), it is of interest the fact that, as compared to control and guadecitabine alone, the association of guadecitabine and ICBs was responsible for amplification of both CD8+ and CD4+ T cell subsets highly expressing IFN-γ, sign of functional activation (Fig. 3C). Collectively, this panel of data suggests that guadecitabine/ICBs impact on the composition of the T cell infiltrate leading to activation and maturation of effector/cytotoxic T cell subsets through mechanisms partly complementary between the two type of agents. Finally, guadecitabine/ICBs did not affect the expression of the alternative immune checkpoint TIM-3 on both CD4+ and CD8+ T cells, whereas ICBs alone strongly upregulated TIM-3, thus potentially driving immune escape (Fig. 3D). Guadecitabine induced an increase in CTLA-4 and PD-1 expression on T cells yet this reached statistical significance only for CTLA-4 on CD4+ T cells, as compared to control (Fig. 3D). The immunostimulatory activity exerted by the combination of guadecitabine/ICBs assumes even more relevance considering that it induced a significant decrease of CD4+CD25+FoxP3+ Treg cells in the tumor infiltrate, indicating that the net effect of this combination treatment is to shift the balance between effector and regulatory T cell functions toward the former one (Fig. 4A).

Guadecitabine/ICBs, in fact, reduced the percentages of immunosuppressive CD4+CD25+FoxP3+ Treg cells compared to any other treatment (Fig. 4A). Furthermore, as compared to control, guadecitabine alone induced a remarkable reduction of myeloid derived suppressor cells (MDSC), in particular the monocytic subset (Ly6C+Ly6G- CD11b+, M-MDSC) (Fig. 4A and Supplementary Fig. 2B), associated with a significant increase of macrophages (Fig. 4B and Supplementary Fig. 2C). Interestingly, combination treatment with guadecitabine/ICBs determined a significant increase of M1 (CD38+Egr2-) macrophages, respect to control and ICBs, indicating that triple therapy skews towards pro inflammatory anti-tumor TAM-M1 responses (Fig. 4B). Taken together, these data indicate that mice receiving triple therapy underwent a shift from an immune suppressive to an immune responsive TME.

### Guadecitabine/ICBs enhance T and NK anti-tumor functions in lymphoid organs, upregulate Th1 responses, and downregulate angiogenic chemokines

**Fig. 5:**
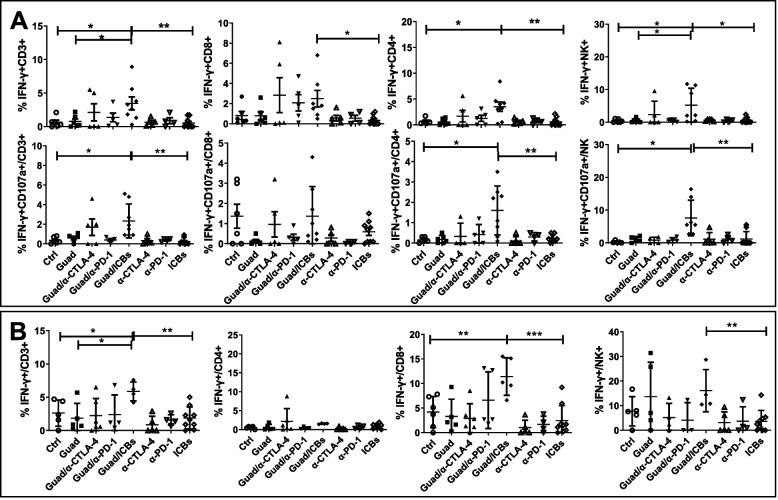
Guadecitabine/ICBs stimulate IFN-γ production and cytotoxic activity on T and NK cells. **A** functional assay on spleen cells from different groups of treatments. Upper row: IFN-γ expression on CD3+, CD4+, CD8+ T and NK cells. Lower row: co-expression of CD107a (marker of degranulation and cytotoxic activity) and IFN-γ on CD3+, CD4+, CD8+ T, and NK cells. **B** functional assay on tumor-draining lymph node cells from different groups of treatments. Percentages of CD3+ IFN-γ+, CD4+ IFN-γ+, CD8+ IFN-γ+ and NKp46+ IFN-γ+ NK cells. *p<0.05, **p<0.02, ***p<0.01

To assess signs of functional activation and commitment to cytotoxic function in lymphoid organs we analyzed IFN-γ and CD107a expression on T and NK cells from spleens and tumor-draining lymph nodes. Guadecitabine/ICBs significantly expanded the frequencies of T and NK cells producing IFN-γ in both spleens and tumor-draining lymph nodes, effect associated with an increased frequency of CD107a^+^ T (Cytotoxic T lymphocytes, CTLs) and NK cells in the spleen, as compared to control and ICBs (Fig. 5 and Supplementary Fig. 3).

**Fig. 6:**
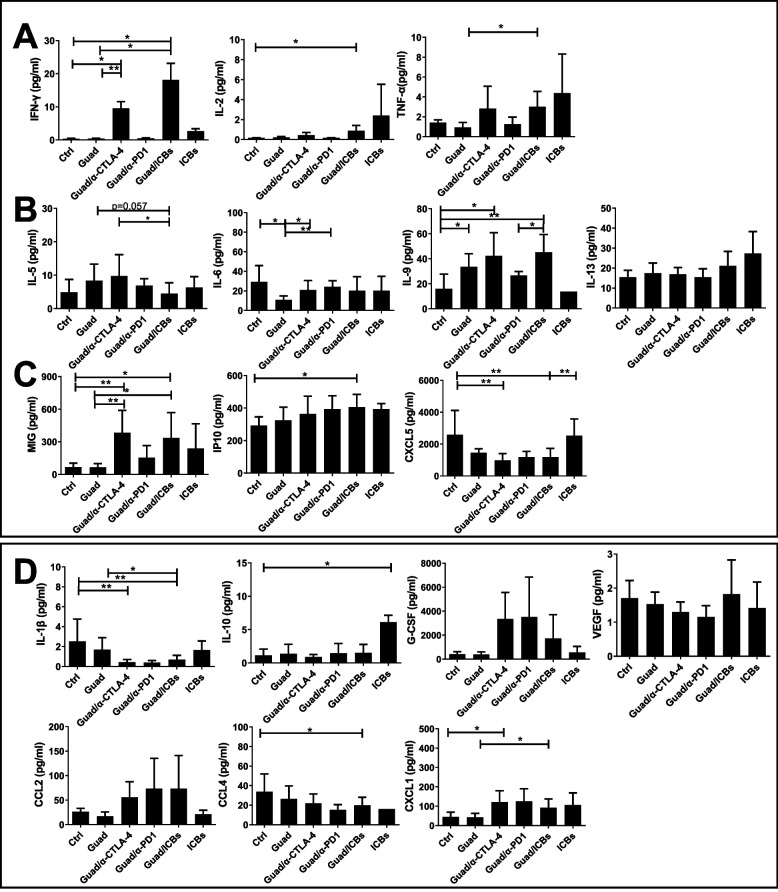
Guadecitabine/ICBs shift towards a Th1 response and inhibit angiogenesis and metastatization. Cytokines level expressed in pg/ml detected by Milliplex assay correlated to: **A** Th1 responses, **B** Th2 responses, **C** angiogenesis and metastasization (CXCL5) regulation and **D** upper row: MDSC generation and activation, **D** lower row: MDSC and leukocyte migration (*n*=3-5 mice/group). *p<0.05, **p<0.02

To corroborate these data we evaluated systemic modifications of cytokine and chemokine levels in the sera from treated and control mice at sacrifice. A strong increase of Th1 cytokine levels, such as IFN-γ, TNF-α and IL-2, was observed in the serum of mice treated with guadecitabine/ICBs in respect to control mice or mice treated with guadecitabine alone (Fig. 6A). Among Th2 cytokines, only IL-9 was up regulated by all guadecitabine containing therapies compared to control, except for guadecitabine/anti-PD-1. IL-5, IL-6, and IL-13 were not modified in guadecitabine/ICBs compared to control (Fig. 6B). The systemic increase of IFN-γ observed in mice treated with guadecitabine/ICBs and guadecitabine/anti-CTLA-4 is followed by an increase of IFN-γ-dependent anti-angiogenic factors, such as MIG and IP10 (Fig. 6C). CXCL5 is a chemokine endowed with angiogenetic and pro-metastatic properties, frequently overexpressed in human cancers where it is considered as a prognostic biomarker [44]. Mice treated with guadecitabine/ICBs and guadecitabine/α-CTLA-4 showed a significant decrease of this chemokine in sera in respect to control mice or mice treated with ICBs (Fig. 6C). TNF-α and G-CSF serum levels were upregulated in mice treated with guadecitabine/ICBs, though not in a statistically significant manner when compared to control mice (Fig. 6D), VEGF and IL-10 were detected at levels similar to those observed for control mice, while IL-1β was significantly lower in mice treated with guadecitabine/ICBs and guadecitabine/α-CTLA-4, compared to control or guadecitabine alone (Fig. 6D).

The serum levels of CCL2 and CCL4, chemokines known to stimulate leukocyte and MDSC migration, were respectively up and down regulated in mice receiving guadecitabine/ICBs compared to control (Fig. 6D). CXCL1 levels increased in mice receiving guadecitabine/anti-CTLA-4 and guadecitabine/ICBs, compared to control and guadecitabine alone, respectively (Fig. 6D).

Comprehensively, these data show that triple therapy can modify not only TME composition, but also T and NK cell responses in lymphoid organs shifting them towards an anti-tumor Th1 cell response.

### Effects of Guadecitabine/ICBs on metastases formation and TME

**Fig. 7:**
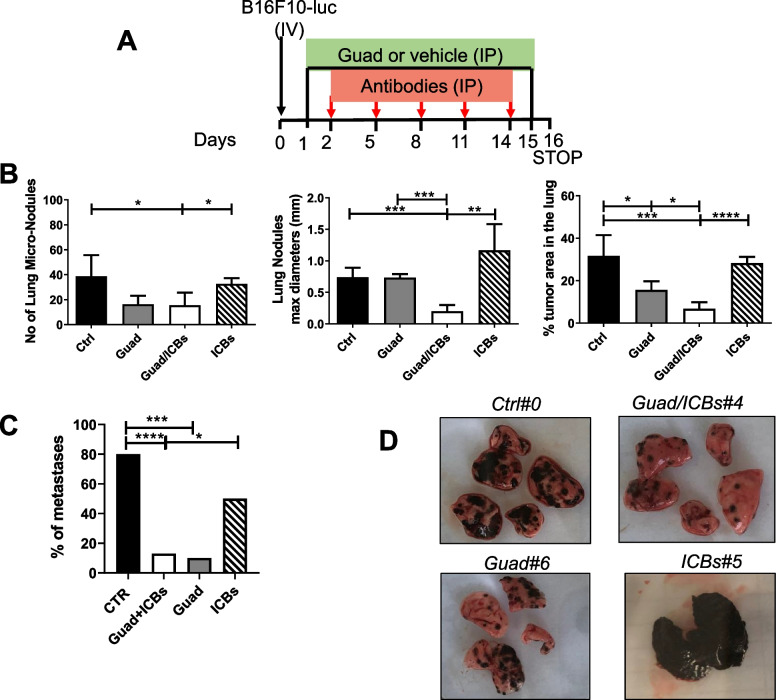
Guadecitabine/ICBs significantly reduce tumor nodules formation in the lung of C57black/6J mice. **A** schedule of treatments: B16F10 cells were injected IV on day 0 (black arrow), guadecitabine or vehicle were given IP daily from day +1 to day +15 (black line), ICBs or Isotype controls (Antibodies) were given IP on days +2, +5, +8, +11, +14 (red arrow). **B** Mice lungs were FFPE and mounted on microscope slides. The histograms show the comparison among each group of treatment concerning: the numbers of lung micronodules (left panel), the mean of nodules maximum diameters (middle panel) and the percentage of lung area occupied by tumor (right panel) (Sample sizes: Ctrl *n*=6, guadecitabine *n*=3, guadecitabine/ICBs *n*=6, ICBs *n*=3). **C** The histogram shows the percentages of mice bearing extra-lung metastases for each group of treatment (Sample sizes: Ctrl *n*=15, guadecitabine *n*=10, guadecitabine /ICBs *n*=15, ICBs *n*=10). **D** Representative images of lungs for each group of treatment. *p<0.05, **p<0.02, ***p<0.01, ****p<0.001

To assess the ability of guadecitabine/ICBs to reduce the development of metastases, we performed an *in vivo* experiment in which C57black/6J mice, injected IV with 4x10^5^ B16F10 cells, received daily IP treatment with guadecitabine (1mg/kg) starting from the day after tumor challenge until day +15. ICBs were administered IP at days +2, +5, +8, +11, +14 post IV challenge. Mice were sacrificed on day 16 and lungs were analyzed for tumor nodules formation (Fig. 7A). Lungs were analyzed for the numbers of micro metastases, the diameters of tumor nodules and the lung area occupied by the tumors. As shown in Fig. 7B the numbers of lung micro metastases were significantly reduced in mice receiving guadecitabine/ICBs compared to control or to mice receiving ICBs alone. The mean maximum diameters of tumor nodules as well as the relative and absolute lung area occupied by the tumors were significantly lower in lungs of guadecitabine/ICBs treated animals, compared to any other treatment (Fig. 7B and D). Guadecitabine containing treatments significantly reduced the number of mice presenting extra-lung metastases, in particular metastases in the peritoneum and the spleen, in respect to control mice. Mice treated with guadecitabine/ICBs also showed a reduction of extra-lung metastases over those treated with ICBs alone (Fig. 7C).

**Fig. 8:**
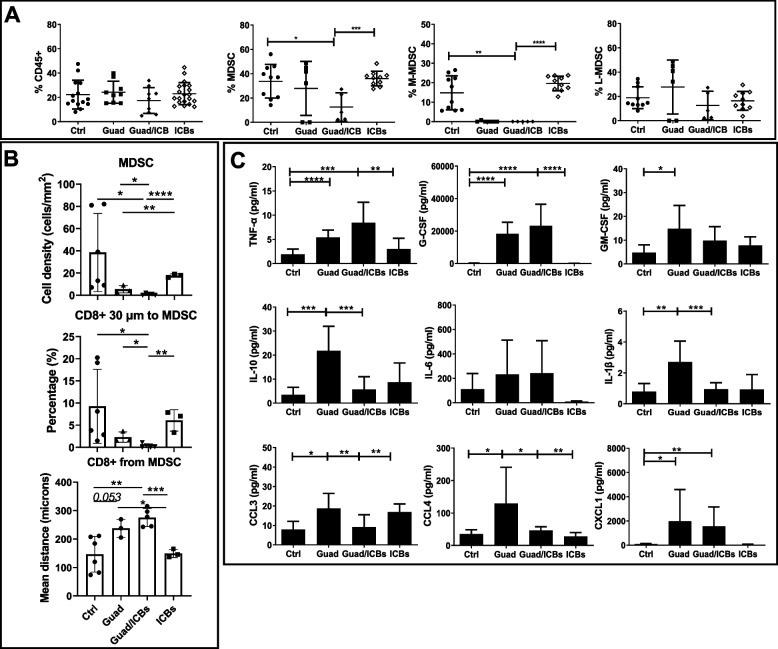
Effects of guadecitabine /ICBs on the percentages of MDSC in proximity to CD8+T cells and on cytokines/chemokine serum levels. **A** percentages of CD45+, MDSC and subsets: M-MDSC (Ly6C+Ly6G-/CD11b+) and G-MDSC (Ly6C^low^Ly6G+/CD11b+) in the lung from mice receiving different treatments. **B** mIF analysis of lung tumor tissue slides (*n*=6 mice/group) showing MDSC cell density (upper histogram), percentages of CD8+ T cells and MDSC (F480-Ly6G+Ly6C-) in a radius distance of 30µm (middle histogram), and mean distance between CD8+ T cells and MDSC (lower histogram) from mice receiving different treatments. **C** serum levels expressed in pg/ml of different cytokines (upper and middle row) or chemokines (lower row) analyzed by Milliplex assay (*n*=7 mice/group). *p<0.05, **p<0.02, ***p<0.01, ****p<0.001

Lungs from control mice, and from mice treated with guadecitabine, ICBs or guadecitabine/ICBs were dissociated into single cell suspensions and studied by flow cytometry to detect treatment induced modifications of TME composition. ICBs treatment increased CD3+ T cell percentages, mainly CD8+ T cell subset, while guadecitabine and guadecitabine/ICBs treatments did not significantly impact on these cell populations, compared to control (Supplementary Fig. 4). Similarly to what observed in the SC tumors, guadecitabine/ICBs significantly reduced total MDSC population frequency in lung metastatic lesions, an effect likely due to the synergic activity of the two treatments since not replicated by guadecitabine or ICBs alone. In particular, M-MDSC were completely depleted by guadecitabine and guadecitabine/ICBs (Fig. 8A). mIF allows the simultaneous visualization and quantification of several antigens on single FFPE tissue sections, maintaining tissue architecture and morphology [45]. Applying mIF on lung tumor tissue slides we could confirm the significant reduction of MDSC. Also, we observed that very few CD8+ T cells were in proximity (within a radius of 30µm) to MDSC and that the percentage of these CD8+ T cells was significantly different between guadecitabine and guadecitabine/ICBs, the latter showing the lowest percentage. The mean distance between CD8+ T cells and MDSC was higher in guadecitabine and guadecitabine/ICBs groups of treatment compared to control or ICBs (Fig. 8B). Reduction of MDSC in guadecitabine/ICBs group was associated with an increase of systemic concentrations of cytokines involved in MDSC and myeloid cell generation such as TNF-α and G-CSF. Guadecitabine treatment increased GM-CSF, IL-10 and IL-1β serum levels compared to control mice or mice treated with ICBs alone. However, IL-10 and IL-1β levels were lower in mice treated with guadecitabine/ICBs in respect to guadecitabine. CCL3, CCL4 and CXCL1 (chemokines involved in leukocyte trafficking, including that of DC and MDSC) were significantly increased upon guadecitabine treatment, while in mice treated with guadecitabine/ICBs CCL3 and CCL4 serum levels were comparable to those of controls (Fig. 8C).

**Fig. 9:**
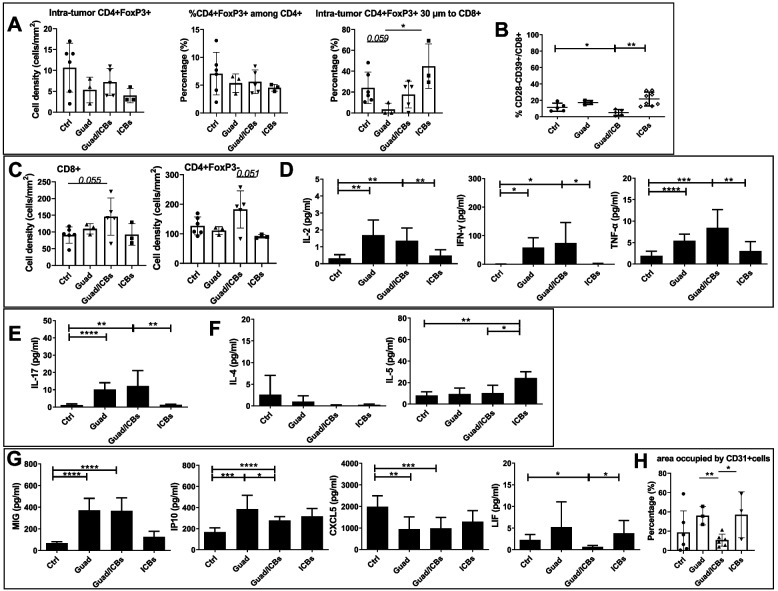
Guadecitabine/ICBs reduce the percentages of immune suppressive T cells more efficiently than guadecitabine alone, increase IFN-γ production and reduce angiogenesis. **A** mIF analysis of lung tumor tissue slides (*n*=3-6 mice/group) showing cell densities of CD4+Foxp3+ regulatory cells, percentages of CD4+FoxP3+ among total CD4+ T cells and percent of CD4+Foxp3+ cells in 30µm proximity to CD8+ cells in tumor nodules from mice receiving different treatments. **B** percentages of CD8+CD28-CD39+ cells in the lung from mice receiving different treatments. **C** mIF analysis of lung tumor tissue slides (*n*=3-6 mice/group) showing CD8+, and CD4+FoxP3- cell densities from mice receiving different treatments. **D** serum levels expressed in pg/ml of Th1 cytokines in response to different *in vivo* treatments by Milliplex assay (*n*=7 mice/group). **E** serum levels expressed in pg/ml of IL-17 cytokine in response to different *in vivo* treatments by Milliplex assay (*n*=7 mice/group). **F** serum levels expressed in pg/ml of Th2 cytokines in response to different *in vivo* treatments by Milliplex assay (*n*=7 mice/group). **G** serum levels expressed in pg/ml of chemokines involved in angiogenesis regulation in response to different *in vivo* treatments by Milliplex assay (*n*=7 mice/group). **H** mIF analysis of lung tumor tissue slides (*n*=3-6 mice/group) showing the percentage of area occupied by CD31+cells from mice receiving different treatments. *p<0.05, **p<0.02, ***p<0.01, ****p<0.001

**Fig. 10:**
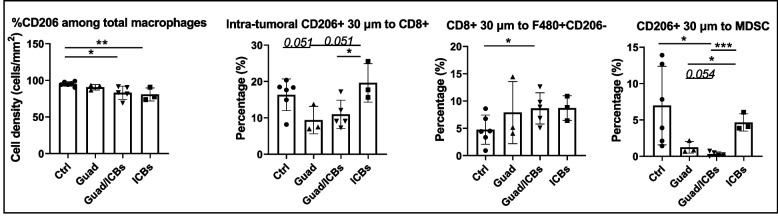
Guadecitabine/ICBs reduce TAM-M2 percentages among total macrophages. mIF analysis of lung tumor tissue slides (*n*=3-6 mice/group). Histograms show, starting from left side, the frequency of CD206+ among total macrophages, percent of CD206+ cells in 30µm proximity to CD8+ cells, the frequency of CD8+ cells within 30µm distance to macrophages CD206- and the frequency of CD206+ cells within 30µm distance to MDSC in the lung from mice receiving different treatments. *p<0.05, **p<0.02, ***p<0.01, ****p<0.001

Immune regulatory CD4+FoxP3+ Treg cells were identified by mIF and their relative prevalence in the lung TME was not significantly affected by any treatment. It is of note that significantly higher percentages of CD4+FoxP3+ Treg were found in close proximity to CD8+T cells in lungs from mice receiving ICBs, compared to those receiving guadecitabine (Fig. 9A), suggesting a possible suppressive effect on CD8+T cells in the ICBs group of treatment. CD8+CD28-CD39+ T cells that have been reported to be immune regulatory and exhausted lymphocytes [46] were significantly decreased upon treatment with guadecitabine/ICBs (Fig. 9B), while total CD8+ and CD4+FoxP3- T cells were increased, as compared to control and ICBs (Fig. 9C). Th1 cytokines (IL-2, IFN-γ, TNF-α) and IL-17, but not Th2 cytokines (IL-4, IL-5) were upregulated in serum samples from mice treated with guadecitabine and its combination with ICBs, as compared to control and ICBs (Fig. 9D, E and F), as well as IFN-γ dependent anti-angiogenic chemokines MIG and IP10 (Fig. 9G). A significative decrease in angiogenesis, determined by a reduction of the area occupied by CD31+ cells in the tumor/peritumor areas, was observed in mice treated with guadecitabine/ICBs compared to guadecitabine or ICBs alone (Fig. 9H). The serum levels of angiogenic chemokines CXCL5 and LIF were significantly diminished by treatment with guadecitabine/ICBs, compared to control and ICBs alone (Fig. 9G). Notably, besides its angiogenic properties, LIF is known to regulate CD206 (a marker for TAM-M2 cell polarization) and to prevent CD8+ T cell tumor infiltration, compromising responses to anti-PD-1 therapy [47]. mIF analysis also revealed that among F4/80+ macrophages those expressing CD206 were significantly decreased in mice receiving guadecitabine/ICBs or ICBs alone, compared to control, and that significantly fewer F4/80+CD206+ M2 cells were close to CD8+T cells in a radial distance of 30µm in lung metastases from mice treated with guadecitabine/ICBs, compared to those treated with ICBs alone. Accordingly, lung tumors from mice treated with triple therapy showed higher percentages of CD8+ T cells close to F4/80+CD206- M1-type macrophages, compared to lung nodules from control mice. Finally, mIF analysis indicated that the percentages of CD206 expressing cells close to MDSC were higher in lung metastases of control mice as well as in mice treated with ICBs, than in mice treated with guadecitabine containing combinations (Fig. 10 and Supplementary Fig. 5A, B).

**Fig. 11:**
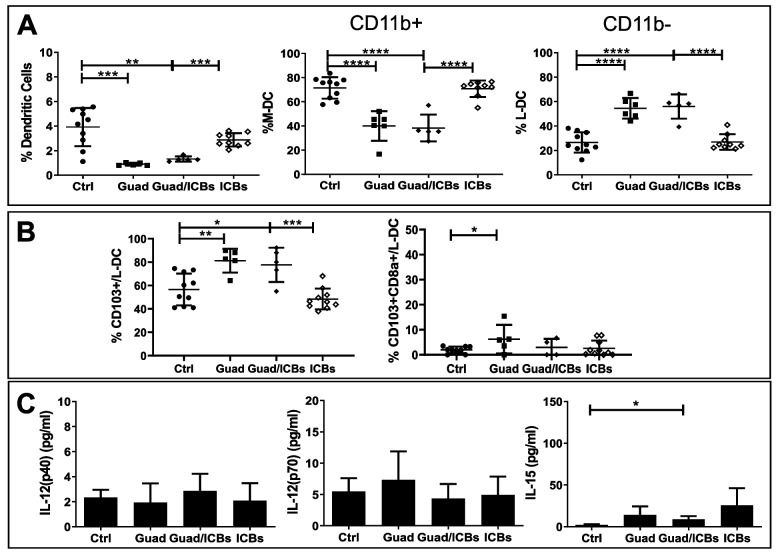
Guadecitabine decreases Dendritic Cells in lung TME, shifting to the subset of Lymphoid-DC. **A** Percentages of Dendritic Cells (CD11c+IAb+) referred to myeloid cells, not T, B, NK, myeloid (M)- (CD11c+IAb+CD11b+), and lymphoid (L)-DC (CD11c+IAb+CD11b-) referred to DC in the lung from mice receiving different treatments by flow cytometry. **B** percentages of L-DC/conventional DC expressing CD103 and co-expressing CD103 and CD8a in the lung from mice receiving different treatments. **C** serum levels expressed in pg/ml of DC cytokines in response to different in vivo treatments by Milliplex assay (*n*=7 mice/group). **p<0.02, ***p<0.01, ****p<0.001

Total DC percentages in lung TME were affected by guadecitabine containing treatments resulting remarkably decreased, compared to control and ICBs (Fig. 11A). However, myeloid- (CD11c+IAb+CD11b+) and lymphoid- (Conventional, CD11c+IAb+CD11b-) DC subsets [48] were regulated by guadecitabine in opposite ways: M-DC were significantly reduced, while L-DC were increased, compared to control mice (Fig. 11A). Guadecitabine, but not ICBs, increased the percentages of CD103+CD11b- and CD103+CD8a+ conventional DC populations, respect to control (Fig. 11B). Interestingly, these cells are highly specialized in priming CD8+ T cells independently from their cross-presentation potential, and produce MIG and IP10 chemokines able to attract T and NK cells [49–52]. Serum levels of IL-12 were not affected by any treatment, while IL-15 levels were increased in serum from mice receiving guadecitabine/ICBs, compared to control (Fig. 11C).

### Guadecitabine determines a significant reduction of DNA-methylation in experimental tumors

**Fig. 12:**
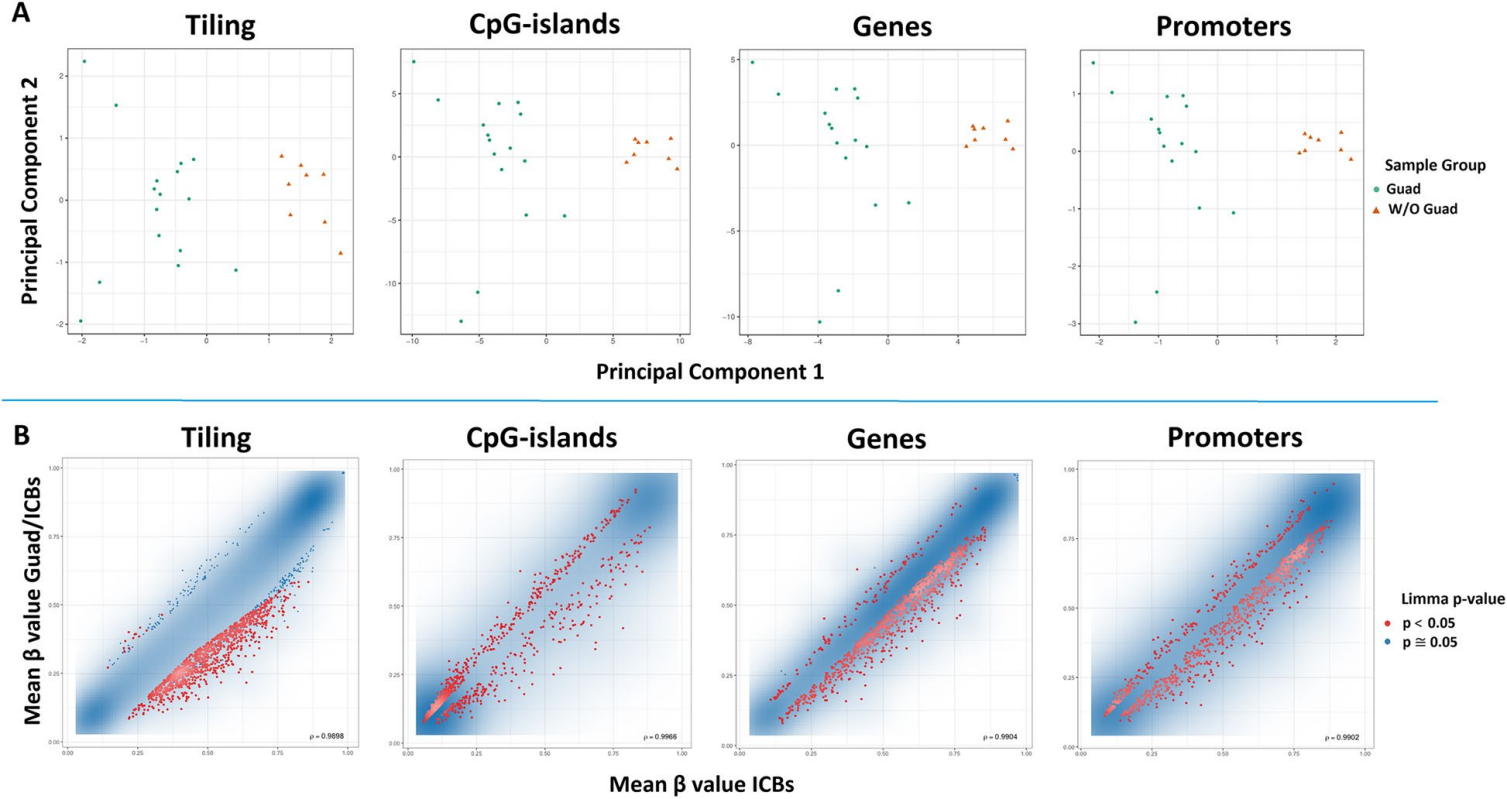
Whole genome methylation analysis of tumors. **A** Scatter plot showing the samples’ coordinates on principal component analysis for all conditions, treatments containing guadecitabine (Guad) = green dots, treatments without guadecitabine (W/O Guad) = ochre triangles. The two treatment conditions are clearly separated by the first two principle components. **B** Scatterplots are shown for samples treated with ICBs alone or triple therapy analyzed by hybridization to Infinium Mouse Methylation BeadChip arrays (Illumina) according to the best combined ranks of signals (scale: mean beta values) derived from all methylation sites (tiling), CpG-islands, and genomic regions containing genes and promoter regions. Blue clouds contain the majority of single methylation sites that are not differentially methylated in a statistically significant manner, red dots indicate sites that are differentially methylated in a significant manner, blue dots correspond to borderline significant sites. Blue clouds show a bimodal distribution of high and low methylation with only a minor population of sites of intermediate methylation, especially so for CpG-islands and promoters

DNA samples isolated from experimental tumors from mice treated with ICBs alone or triple therapy were analyzed by hybridization to Infinium Mouse Methylation BeadChip arrays and analyzed for all methylation sites (“tiling”) or signals derived from CpG-islands, genomic regions containing genes and promoter regions of protein coding genes. Principal component analysis (PCA) of samples exposed to guadecitabine as opposed to those not exposed showed that these two sample types are clearly distinct, irrespective of additional treatments with ICBs (Fig. 12 A).

DNA-methylation showed the typical bimodal distribution of high and low methylation with only a minor population of sites of intermediate methylation, especially so for CpG-islands and promoters. Partial pharmacological inhibition of DNA Methyltransferase 1 (DNMT1) in the tumors of mice treated with the combination of guadecitabine and ICBs as compared to those treated with ICBs alone, determined limited effects on highly methylated sites (sites methylated in most or all cells). The drug mainly affects sites of intermediate methylation. As expected, the addition of guadecitabine to the two ICBs determined a reduction of methylated sites, in particular for promoters and genes and less so for CpG-islands (Fig. 12 B).

**Fig. 13:**
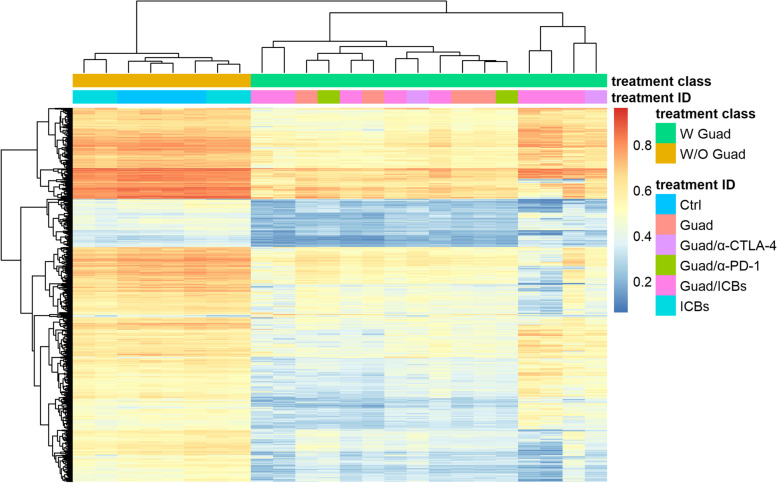
Heatmap of the 1% most variable methylation probes in terms of median absolute deviation (MAD). Probes methylated above mean are reported in red, below mean in blue; the color intensity indicates the distance from mean values. Samples are annotated with two color bars at the top of the heatmap (treatment class): green=with Guad , gold=without Guad. The second color bar (treatment ID) reports mouse treatment as Control (Ctrl), Guad alone or combined with one (Guad/α-CTLA-4, α-PD-1) or both immune check-point inhibitors (Guad/ICBs). Clusters defined by the Pheatmap package [37] are reported by the dendrogram on top of the heatmap

PCA (Fig. 12A) showed clear differences between samples obtained from treated with and without guadecitabine. In consistence with PCA, class comparison analysis of these two groups revealed that they form two main clusters of distinct methylation patterns (Fig. 13).

**Fig. 14:**
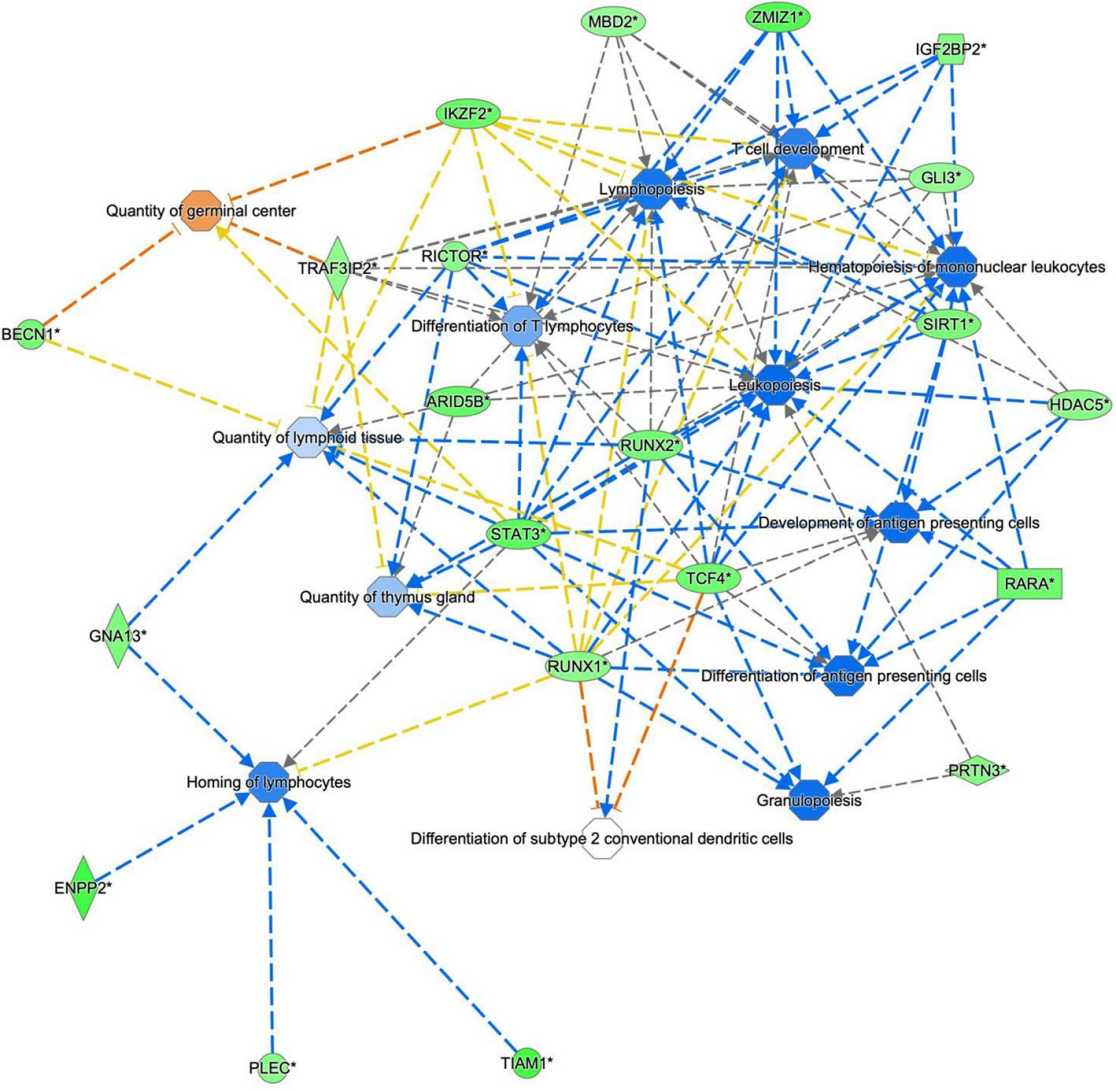
Significant biological networks generated by differentially methylated probes filtered in terms of MAD based on the connectivity of the genes including the methylation probes. The network is constituted by genes involved in the lymphoid tissue structure and development and in the homeostasis of the immune system. All genes identified are hypomethylated by Guad treatment and are predicted to activate several processes of the immune system represented by trapezoidal shapes. Green genes represent demethylated genes, blue hexagonal shapes represent biological processes predicted to be activated by demethylated genes and orange hexagonal shapes those predicted to be inactivated

The analysis of the DNA methylation data, using the most variable 1% of probes, identified 103 probes (mean difference < 0.16; *p* <0.01), classifying 334 genes for different transcript/isoforms differentially methylated between tumors arising from guadecitabine-treated mice contrasted with guadecitabine-untreated ones (Fig. 13 and Supplementary Table 1). The pathway analysis of these differentially methylated genes highlighted significant immune system related biological networks, among these T cell development, differentiation, and antigen presentation (Fig. 14).

## Discussion

Immune checkpoint blockers (ICBs) have dramatically improved survival after diagnosis of metastatic melanoma, yet most patients progress during therapy or relapse after initial response [5, 6]. Guadecitabine is a demethylating agent resistant to cytidine deaminase that acts through the inhibition of DNA Methyltransferase 1 (DNMT1), causing non-specific hypomethylation. Guadecitabine induces a strong up-regulation of HLA-class I antigens and of Intercellular Adhesion Molecule-1 (ICAM-1), thus being an optimal partner to improve the therapeutic efficacy of immunotherapeutic agents [53]. Accordingly, combination of guadecitabine with antibodies directed against CTLA-4 (ipilimumab) has already been tested in human melanoma patients (NIBIT-M4 trial, NCT02608437). In this trial, guadecitabine revealed to be safe and well tolerated showing promising immunomodulatory and antitumor activity [20]. The next step, the association of guadecitabine with anti-CTLA-4 and anti-PD-1 mAbs, is being explored in an ongoing clinical phase 2 trial (NIBIT-ML1, NCT04250246).

Here, we investigated the mechanisms and the immune cells involved in the response to a triple combination therapy including guadecitabine and two ICBs (anti-CTLA-4 and anti-PD-1 mAbs) in a SC and a pseudo-metastatic syngeneic mouse model of melanoma.

Our data indicate that guadecitabine alone reduced the growth of SC tumors and of metastases in the pseudo-metastatic model. More importantly, the addition of guadecitabine potentiated the effects of anti-CTLA-4 and anti-PD-1 mAbs. The triple combination was also superior to all other treatment conditions in terms of tumor growth control and immune modulatory effects on tumor infiltrating lymphocytes. In addition, triple therapy significantly reduced the number of extra-lung metastases, in the pseudo-metastatic model.

SC tumors developed upon B16F10 injection are in general cold tumors, only slightly sensitive to ICBs treatment, with B16F10 presenting low MHC class I cell surface expression levels due to the impaired expression of antigen presenting machinery components, that has already been reported to be restored by treatment with IFN-γ and DNMT inhibitors [54, 55]. Our data demonstrate that *in vivo* treatment with guadecitabine increased the expression of MHC-class I on B16F10 cells, and that its combination with ICBs further enhanced this upregulation, likely contributing to a better tumor control. An important antitumor effect of guadecitabine is related to the inhibition of the accumulation and/or differentiation of immune suppressive MDSC in the TME. Total MDSC appear depleted in mice receiving treatment with guadecitabine/ICBs, in agreement with Luker et al. who reported similar results in an *in vivo* model of breast cancer [56]. Guadecitabine increased serum levels of TNF-α, GM-CSF, G-CSF, IL-1β, IL-10, factors inducing MDSC [57], and CCL3 and CXCL1, chemokines involved in the migration of M-MDSC [58, 59] in the pseudo-metastatic model, possibly inducing a sort of feedback mechanism.

Guadecitabine/ICBs, differently from ICBs alone, did not up-regulate the alternative immune checkpoint molecule TIM-3 on T cells, thus preventing this mechanism of tumor escape and exhaustion [60]. Treatment with guadecitabine/ICBs down modulated the percentages of CD4+ Tregs in the SC model of melanoma. The effect of demethylating agents on CD4+ Treg cells has been investigated with contrasting results. Some authors referred that Treg cells are enhanced by azacytidine during inflammatory conditions in mouse models thus preserving the animals from viral progression [61], or that decitabine-treated conventional T cells acquired the ability to produce FoxP3 and to mediate suppressor functions [62–64]. Other authors reported that the deletion of DNMT1 in mice decreased the number of Treg cells in lymphoid organs [65] and that patients on trial with guadecitabine and anti-PD1 (pembrolizumab) showed a reduced number of Treg cells compared to baseline in tumor biopsies [66]. Beyond CD4+ Tregs, also CD8+ T cells display immune regulatory activities [46, 67]. Our data reveal that guadecitabine/ICBs significantly down modulated CD8+CD28-CD39+ T cells in the pseudo-metastatic model, thus further limiting immune suppression. In the SC model, ICBs increased the percentages of CD8+CD28-CD39+ T cells, while the addition of guadecitabine to ICBs reduced this population. CD8+ CD28− T cells are a heterogenous group of cells comprising bona fide regulatory cells, senescent and exhausted T cells [67]. Their tolerogenic activity was observed in the model of experimental colitis, inflammatory bowel disease and in experimental autoimmune encephalitis (EAE) [67], suggesting that targeting CD8+CD28- Tregs or their depletion may represent a potential strategy to enhance antitumor immunity in cancer. These cells express CD39 that is involved in the production of immunosuppressive adenosine [38–40] and considered a novel potential target for drugs in cancer immunotherapy [68]. In human head and neck cancer, the presence of CD8+CD28-CD127-CD39+ Treg also expressing markers of exhaustion, was found in poor responders to treatment [69]. To the best of our knowledge, the present is the first study to report a significative effect of guadecitabine/ICBs on CD8+ Tregs down modulation in mice.

Guadecitabine stimulated CD8+ T cell maturation towards the effector memory stage and allowed them to acquire cytotoxic activity through the production of granzyme. But only triple therapy induced T cells to secrete IFN-γ in the TME as well as to degranulate and produce IFN-γ in lymphoid organs, suggesting the presence at these sites of effector CTLs or of effector memory CTLs displaying lytic functions. Similarly, NK cells also exhibited cytotoxic functions and produced IFN-γ in lymphoid organs in mice receiving triple therapy. Our data indicate that guadecitabine and guadecitabine/ICBs strongly augmented systemic levels of different Th1 cytokines, except for IL-12. IFN-γ, which is highly produced in response to guadecitabine containing treatments, may drive Th1 differentiation in the absence of IL-12, as previously reported [70, 71]. IFN-γ also induces T-bet (T box expressed in T cells) expression, an important transcription factor expressed early by Th1 cells, that regulates IFN-γ production itself [72, 73]. IFN-γ upregulation in the TME of mice bearing SC melanoma and treated with guadecitabine/ICBs may stimulate TAM-M1 polarization [74], that can be further sustained by the up regulation of IFN-γ and TNF-α systemic levels. M1 macrophage polarization has been linked to an enhanced anti-cancer activity and correlates with favorable prognosis and longer survival [75–77]. Conversely, the accumulation of TAM-M2 in melanoma is a poor indicator of patients’ outcome [78]. In the pseudo-metastatic model, the reduction of serum levels of the cytokine LIF is suggestive of a general reduction of TAM-M2, given the role of LIF in stimulating CD206 expression and M2-cell functions [47]. The reduction of M2 cells among total macrophages was confirmed by mIF analysis in mice receiving ICBs or guadecitabine/ICBs. The residual CD206+ M2 cells in mice treated with ICBs were in close proximity to CD8+ T cells in lung nodules, thus potentially impairing their anti-tumor effector functions. Yet, tumors from guadecitabine/ICBs treated animals displayed a significantly lower amount of CD206+ M2 close to CD8+ T cells, but relatively high percentages of CD206- macrophages, suggestive of the establishment of a productive anti-tumor crosstalk. IFN-γ has strong antitumor functions that also rely on the induction of an anti-angiogenic cascade involving MIG and IP10 (CXCL9 and CXCL10) [79] and on a direct inhibitory effect on tumor cell growth *in vivo* [80]. Triple therapy also induced higher amounts of systemic IFN-γ, MIG and IP10, and reduction of CXCL5 sustaining an anti-angiogenic effect *in vivo*. Indeed, a significative decrease of the area occupied by CD31+ cells in the tumor/peritumor areas, was observed in mice treated with guadecitabine/ICBs compared to guadecitabine or ICBs alone.

Mature DCs link the innate to the adaptive immune system through their unique functions [81, 82]. However, there are several DC subsets provided with different capacity to induce a cytotoxic immune response [48–51]. Interestingly, in our SC and pseudo-metastatic models, guadecitabine reduced the percentages of total DC in the TME but when associated with ICBs shifted the DC population towards differentiation in lymphoid/conventional DC, which are characterized by the absence of CD11b and the expression of CD103 (cDC1). This DC subset is the most effective in inducing CTLs and Th1 responses to tumor cells [49–52]. The observed upregulation of serum MIG and IP10 may therefore represent a signal from DC to recruit CD8+ effector T and NK cells into the tumor tissue [50]. Hence, this set of data indicates that guadecitabine is able to select within TME a DC subpopulation able to sustain effector immune responses.

The modification of TME observed in mice treated with triple therapy may in part be related to the demethylating activity of guadecitabine. Samples obtained from mice treated with the DNMT1 inhibitor exhibited a clearly different methylation status as compared to untreated samples. Guadecitabine prevalently demethylated sites in promoters and CpG-islands of intermediate methylation. The lack of effect on sites that are methylated in only few cells or are completely unmethylated is explained by the fact that these sites cannot be further de-methylated. The absence of effect on highly methylated sites might indicate that these sites are completely silenced by methylation in a stable manner. DNMT1 is directed towards replication foci through the interaction of its replication foci targeting sequence domain with the methylated histone H3K9me3 [83] whose distribution in the genome might not be uniform. Persistent methylation despite partial inhibition of DNMT1 could be due to an abundance of the enzyme in heterochromatin where it is enriched through interaction of its bromo-adjacent-homology domain with histone H4 methylated at the lysine residue 20 (H4K20me3) [84]. Treatment with guadecitabine might not be sufficient to alter methylation in the regions of high abundance of the enzyme. Demethylation of DNA is generally associated with increased gene expression. This is particularly true for CpG-islands and promoter regions and much less so for the gene body where demethylation has also been linked to reduced overexpression of oncogenes [85]. Given the random effect of DNMT1 inhibition that is expected to lead to different patterns of methylation in each single cell, we do not expect specific DNA-methylation events to be detected in guadecitabine treated samples. Major effects could be mediated by effector genes in immune cells where the (re-)activation of specific genes in only a minor fraction of cells can induce strong proliferation of effector cells and reversal of T-cell exhaustion [15], that are the major rationale for the addition of epigenetic drugs to ICBs [20, 86, 87]. Indeed, two clusters corresponding to cases treated with and without guadecitabine are clearly evident by the analysis of 1% of probes differentially methylated and the 334 differentially methylated genes impact on significant biological networks, such as T cell differentiation, development, and antigen presentation. These data indicate that guadecitabine may have induced a robust immune activation able of reshaping anti-tumor response.

Even if triple therapy significantly control tumor growth and stimulate anti-tumor responses, all mice developed tumors and none of them resulted cured. We may speculate that alternative immune checkpoints, other than the ones studied, might be upregulated thus pushing T cells in an exhausted state, favoring tumor growth, or that Treg cells, though strikingly diminished by triple therapy, maintain their suppressive functions thus shutting down anti-tumor T and NK functions. One should also remind that the B16F10 tumor model is particularly aggressive, only leaving a short time for therapeutic intervention and scarcely respond to ICB mediated immunotherapy [32].

## Conclusion

Our data demonstrate that guadecitabine synergizes with ICBs in limiting tumor growth by a twofold effect: reducing immune suppressive cell subsets (MDSC and Treg) and inducing a Th1/Tc1 anti-tumor response in both the SC and pseudo-metastatic models of murine melanoma. This work provides preclinical data supporting the addition of epigenetic drugs, such as guadecitabine, to ICBs to increase the number of responding patients and the duration of response.

## Supplementary Information

**Additional file 1: Supplementary Figure 1.** A: analysis of viable CD45+, CD3+, CD4+ and CD8+ cells (referred to live CD45+cells) in tumors from mice receiving different in vivo treatments. No significative differences in percentages of cells were detected among different group of treatments. B: analysis of CD8+CD28-CD39+ regulatory cells percentages in tumors from mice receiving different in vivo treatments. *p<0.05.

**Additional file 2: Supplementary Figure 2.** Gating strategies. A: T cells: tumor infiltrating leukocytes were gated based on SSC-A versus FSC-A and singlets were selected from the FSC-A versus FSC-H dot plot. Dead cells were excluded with Fixable Viability Dye (FVD). On viable cells, CD45+ cells were selected and within this population CD3+T cells. From CD3+ cells CD8+ and CD4+ were differentiated. B: tumor infiltrating leukocytes were gated based on SSC-A versus FSC-A and singlets were selected from the FSC-A versus FSC-H dot plot. Dead cells were excluded with Fixable Viability Dye (FVD). On viable cells, CD45+ cells were selected and among CD45+ cells we studied CD11b+ cells. Representative dot plot of MDSC cell subset: monocytic- (Ly6C+Ly6G-) or granulocytic- (Ly6ClowLy6G+). C: dot plot of myeloid sub population selected on singlet, viable, CD45+ cells as I-Ab-CD11c-/+. Monocytes: Ly6C+Ly6G-, neutrophils: Ly6G+Ly6C+ and macrophages: Ly6C-Ly6G-. On macrophage cell population we analyzed M1/M2 polarization (M1: CD38+Egr2-, M2: CD38-Egr2+).

**Additional file 3: Supplementary Figure 3.** Gating strategies. A: spleen: leukocytes were gated based on SSC-A versus FSC-A and singlets were selected from the FSC-A versus FSC-H dot plot. Dead cells were excluded with Fixable Viability Dye (FVD). CD45+ from spleen cells were separated in CD3+ cells (CD4+ and CD8+) and in CD3-NKp46+ NK cells. B: lymph nodes: leukocytes were gated based on SSC-A versus FSC-A and singlets were selected from the FSC-A versus FSC-H dot plot. Dead cells were excluded with Fixable Viability Dye (FVD). CD45+ cells from tumor draining lymph nodes were separated in CD3+ cells (CD4+ and CD8+) and in CD3-NKp46+ NK cells. Dot plots of CD3+, CD4+, CD8+, NK cells are given.

**Additional file 4: Supplementary Figure 4.** Analysis of viable CD3+, CD4+ and CD8+ cells, referred to live CD45+ cells, in lung tumors from mice receiving different in vivo treatments. *p<0.05, **p<0.02, ***p<0.01.

**Additional file 5: Supplementary Figure 5.** mIF staining of lungs from mice injected IV with B16F10 and treated with guadecitabine, guadecitabine/ICBs, ICBs or ctrl. A: Representative nine-colour multispectral images of a lung sample slide. Original magnification ×20. Immune markers and colour codes are indicated in the legend. B: Representative image of cell-cell distance analysis. CD206+ TAM-M2 cells (light green dots) within a 30 µm radius from CD8+ T cells (red dots) are represented. The distance of 200µm is represented as a reference scale.

**Additional file 6: Supplementary Table 1.** List of the 334 genes identified by 103 probes using the most variable 1% of probes differentially methylated between the tumors from mice treated with guadecitabine compared to those without guadecitabine.

## Data Availability

DNA-methylation microarray data are available under accession number GSE220698 at the GEO (http://www.ncbi.nlm.nih.gov/geo/).

## Abbreviations

Guad: Guadecitabine
ICBs: Anti-CTLA-4 + anti-PD-1
CTR: Control
TME: Tumor microenvironment
TAM: Tumor associated macrophages
MDSC: Myeloid derived suppressor cells
DC: Dendritic cells
GM-CSF: Macrophage colony-stimulating factor
G-CSF: Granulocyte colony-stimulating factor
M-CSF: Macrophage colony-stimulating factor
VEGF: Vascular endothelial growth factor
TNF-α: Tumor necrosis factor-α
mIF: Multiplex immunofuorescence
