# Supplementary figures and images for: Guadecitabine increases response to combined anti-CTLA-4 and anti-PD-1 treatment in mouse melanoma in vivo by controlling T-cells, myeloid derived suppressor and NK cells
Source: J Exp Clin Cancer Res. 2023 Mar 18;42:67. doi: 10.1186/s13046-023-02628-x (PMC10024396; doi:10.1186/s13046-023-02628-x)

**A**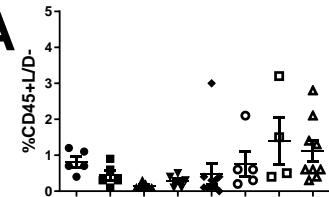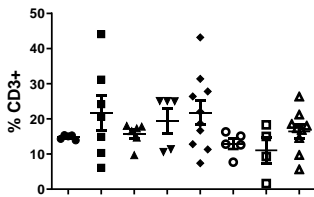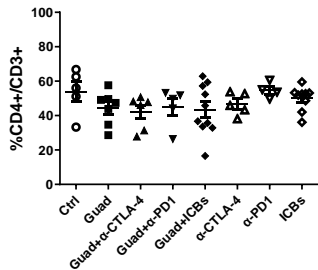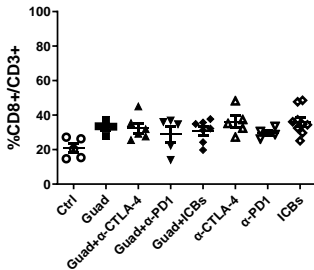**B**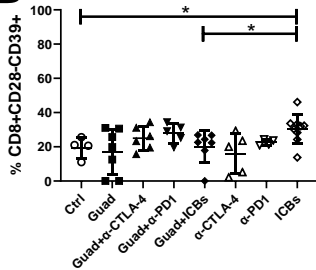

Supplement: Supplementary file 1 — Additional file 1: Supplementary Figure 1. A: analysis of viable CD45+, CD3+, CD4+ and CD8+ cells (referred to live CD45+cells) in tumors from mice receiving different in vivo treatments. No significative differences in percentages of cells were detected among different group of treatments. B: analysis of CD8+CD28-CD39+ regulatory cells percentages in tumors from mice receiving different in vivo treatments. *p<0.05. [file 13046_2023_2628_MOESM1_ESM.pdf]

## A Spleen Gating strategy

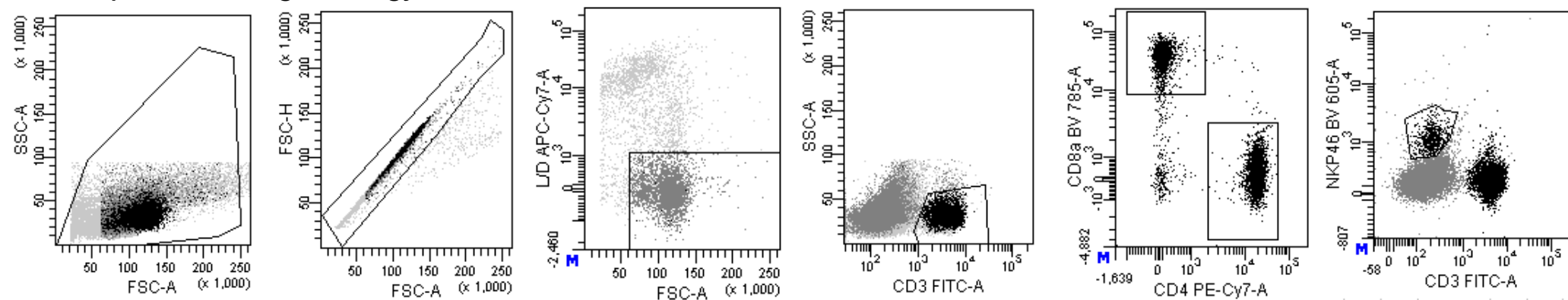

## B LN Gating strategy

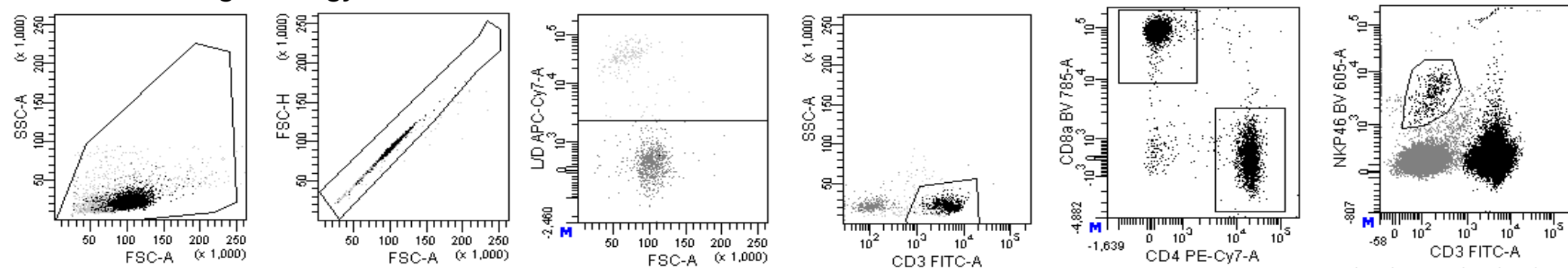

Supplement: Supplementary file 3 — Additional file 3: Supplementary Figure 3. Gating strategies. A: spleen: leukocytes were gated based on SSC-A versus FSC-A and singlets were selected from the FSC-A versus FSC-H dot plot. Dead cells were excluded with Fixable Viability Dye (FVD). CD45+ from spleen cells were separated in CD3+ cells (CD4+ and CD8+) and in CD3-NKp46+ NK cells. B: lymph nodes: leukocytes were gated based on SSC-A versus FSC-A and singlets were selected from the FSC-A versus FSC-H dot plot. Dead cells were excluded with Fixable Viability Dye (FVD). CD45+ cells from tumor draining lymph nodes were separated in CD3+ cells (CD4+ and CD8+) and in CD3-NKp46+ NK cells. Dot plots of CD3+, CD4+, CD8+, NK cells are given. [file 13046_2023_2628_MOESM3_ESM.pdf]

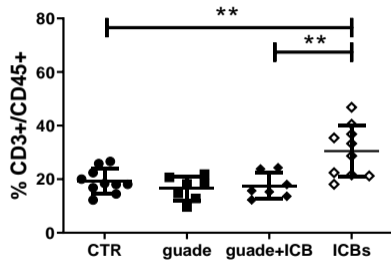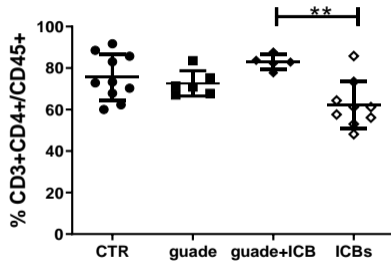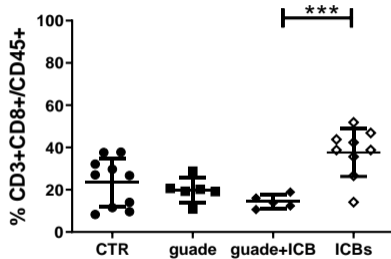

Supplement: Supplementary file 4 — Additional file 4: Supplementary Figure 4. Analysis of viable CD3+, CD4+ and CD8+ cells, referred to live CD45+ cells, in lung tumors from mice receiving different in vivo treatments. *p<0.05, **p<0.02, ***p<0.01. [file 13046_2023_2628_MOESM4_ESM.pdf]
