# Supplementary material for: Guadecitabine increases response to combined anti-CTLA-4 and anti-PD-1 treatment in mouse melanoma in vivo by controlling T-cells, myeloid derived suppressor and NK cells
Source: J Exp Clin Cancer Res. 2023 Mar 18;42:67. doi: 10.1186/s13046-023-02628-x (PMC10024396; doi:10.1186/s13046-023-02628-x)
Supplement: Supplementary file 2 — Additional file 2: Supplementary Figure 2. Gating strategies. A: T cells: tumor infiltrating leukocytes were gated based on SSC-A versus FSC-A and singlets were selected from the FSC-A versus FSC-H dot plot. Dead cells were excluded with Fixable Viability Dye (FVD). On viable cells, CD45+ cells were selected and within this population CD3+T cells. From CD3+ cells CD8+ and CD4+ were differentiated. B: tumor infiltrating leukocytes were gated based on SSC-A versus FSC-A and singlets were selected from the FSC-A versus FSC-H dot plot. Dead cells were excluded with Fixable Viability Dye (FVD). On viable cells, CD45+ cells were selected and among CD45+ cells we studied CD11b+ cells. Representative dot plot of MDSC cell subset: monocytic- (Ly6C+Ly6G-) or granulocytic- (Ly6ClowLy6G+). C: dot plot of myeloid sub population selected on singlet, viable, CD45+ cells as I-Ab-CD11c-/+. Monocytes: Ly6C+Ly6G-, neutrophils: Ly6G+Ly6C+ and macrophages: Ly6C-Ly6G-. On macrophage cell population we analyzed M1/M2 polarization (M1: CD38+Egr2-, M2: CD38-Egr2+). [file 13046_2023_2628_MOESM2_ESM.pdf]

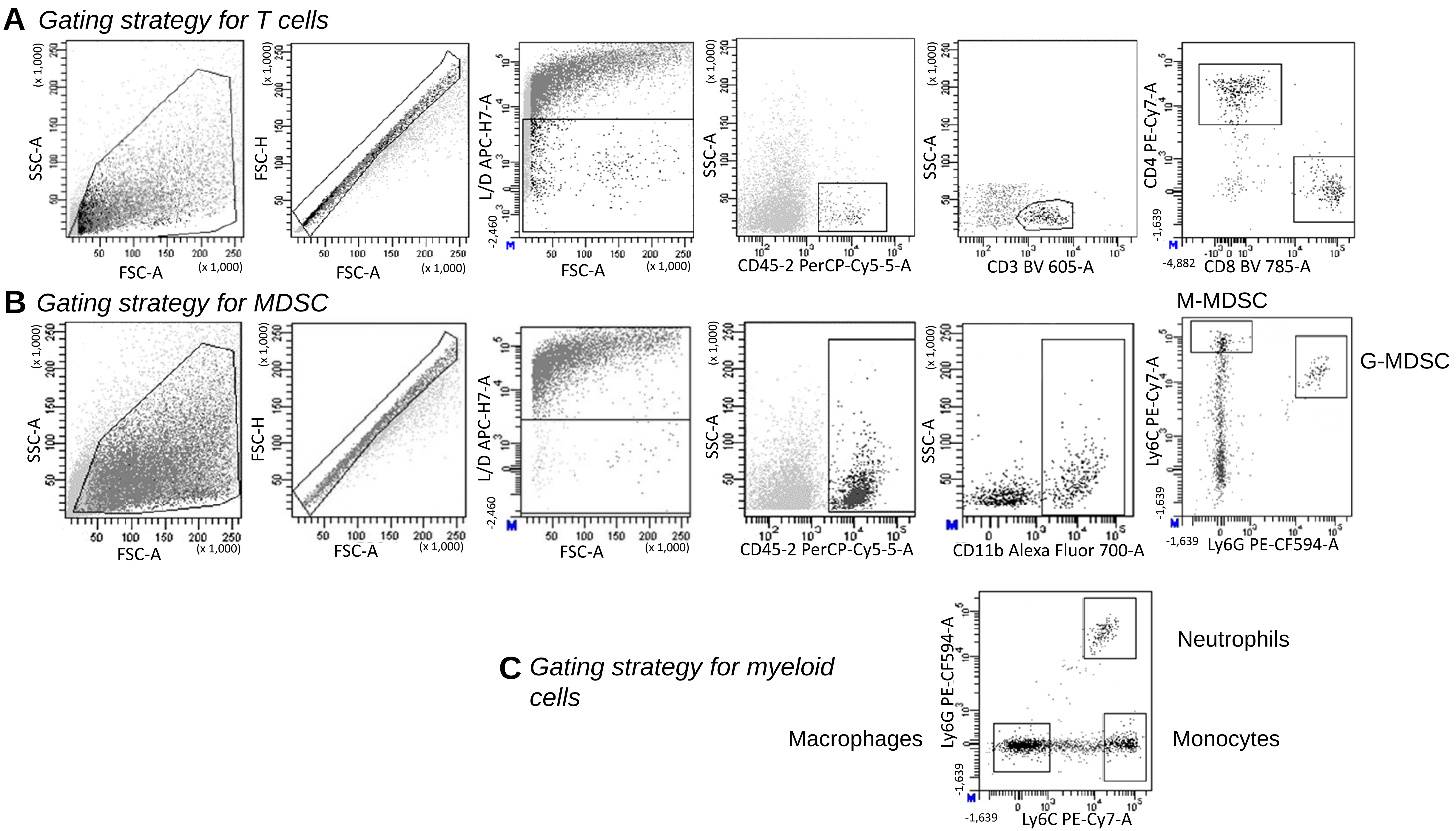
A Gating strategy for T cells
SSC-A
FSC-A
(x 1,000)
FSC-H
L/D APC-H7-A
-2,460
CD45-2 PerCP-Cy5-5-A
CD3 BV 605-A
CD4 PE-Cy7-A
-1,639
-4,882
CD8 BV 785-A
B Gating strategy for MDSC
M-MDSC
G-MDSC
CD11b Alexa Fluor 700-A
Ly6C PE-Cy7-A
Ly6G PE-CF594-A
C Gating strategy for myeloid cells
Neutrophils
Macrophages
Monocytes
