# Supplementary material for: Guadecitabine increases response to combined anti-CTLA-4 and anti-PD-1 treatment in mouse melanoma in vivo by controlling T-cells, myeloid derived suppressor and NK cells
Source: J Exp Clin Cancer Res. 2023 Mar 18;42:67. doi: 10.1186/s13046-023-02628-x (PMC10024396; doi:10.1186/s13046-023-02628-x)
Supplement: Supplementary file 5 — Additional file 5: Supplementary Figure 5. mIF staining of lungs from mice injected IV with B16F10 and treated with guadecitabine, guadecitabine/ICBs, ICBs or ctrl. A: Representative nine-colour multispectral images of a lung sample slide. Original magnification ×20. Immune markers and colour codes are indicated in the legend. B: Representative image of cell-cell distance analysis. CD206+ TAM-M2 cells (light green dots) within a 30 µm radius from CD8+ T cells (red dots) are represented. The distance of 200µm is represented as a reference scale. [file 13046_2023_2628_MOESM5_ESM.pdf]

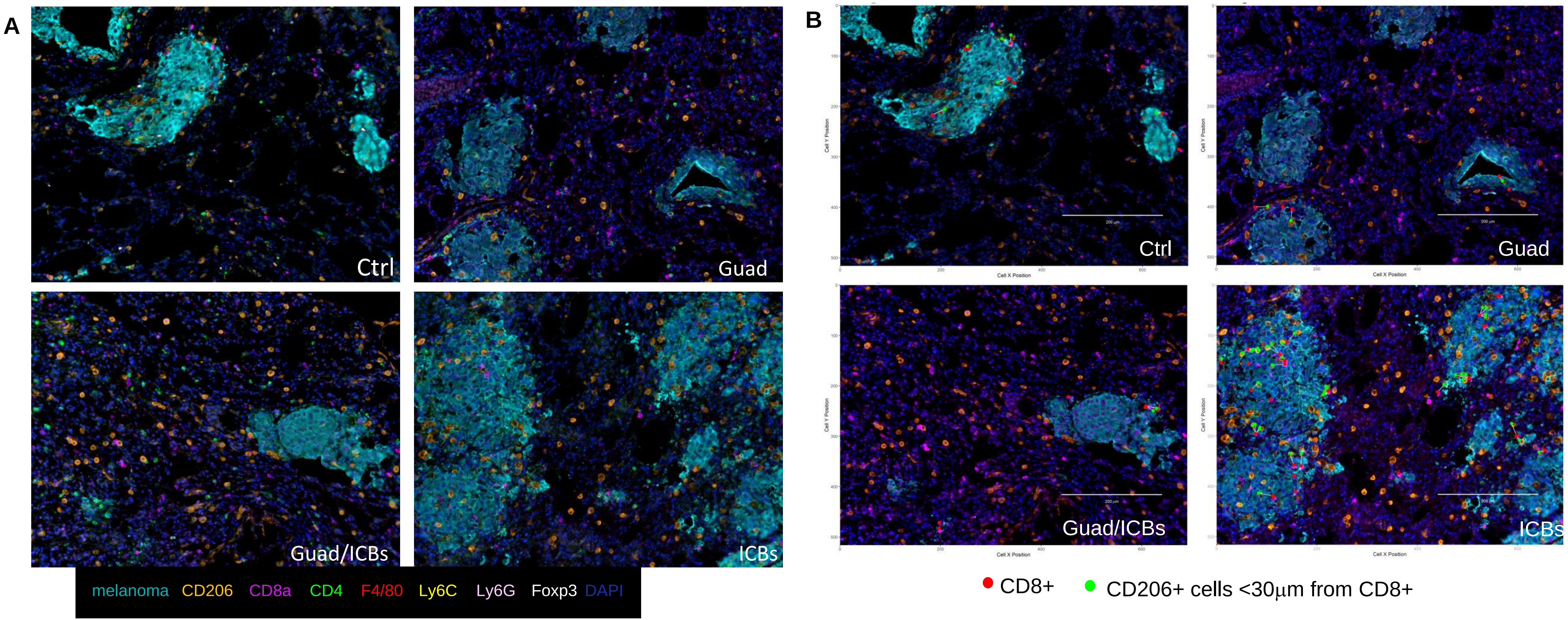
A
Ctrl
Guad
Guad/ICBs
ICBs
melanoma CD206 CD8a CD4 F4/80 Ly6C Ly6G Foxp3 DAPI
B
Ctrl
Guad
Guad/ICBs
ICBs
Cell X Position
Cell Y Position
CD8+
CD206+ cells <30μm from CD8+
